# A psychometrics of individual differences in the truth effect

**DOI:** 10.3758/s13423-026-02975-8

**Published:** 2026-09-23

**Authors:** Selina Zajdler, Martin Schnuerch

**Affiliations:** Chair of Research Methods and Psychological Assessment, School of Social Sciences, University of Mannheim, 68161 Mannheim, Germany

**Keywords:** Truth effect, Individual differences, Reliability, Psychometrics, Signal-to-noise

## Abstract

The truth effect, the tendency to judge repeated information as more truthful, is a robust cognitive phenomenon. Yet efforts to study individual differences in this effect have returned inconsistent results. We argue that these inconsistencies largely reflect methodological limitations. Specifically, since correlations are attenuated by low reliability, conflicting findings likely stem from inconsistent measurement reliability across studies. In this structured, large-scale analysis of truth-effect datasets, we assess the psychometric properties of truth-effect paradigms. Rather than focusing solely on reliability – which depends on the number of replicate trials – we use signal-to-noise ratios, a recently proposed metric that captures psychometric goodness independent of trial size. Together, signal-to-noise ratios and trial size determine reliability, and both vary considerably across studies. Because increasing trial size alone is not always sufficient to ensure adequate reliability, we identify design characteristics that influence psychometric goodness in truth-effect experiments, such as stimulus material, initial task, sample composition, and study setting. Based on these findings, we offer practical recommendations for designing truth-effect experiments that afford the necessary psychometric goodness to measure individual differences. We show that, when psychometric goodness is high, localizing individual differences and their correlations with other psychological constructs is feasible with a manageable number of trials. These findings provide a basis for future research on individual differences in the truth effect and further underscore the importance of psychometric considerations in cognitive experiments.

Hearing a claim repeatedly makes it feel more true, even when it isn’t. This cognitive bias, known as the *truth effect*, may explain why myths like “rowan berries are poisonous”, “the Eskaleut languages know a hundred words for snow”, or “vaccinations cause autism” persist despite being factually incorrect. The effect of repetition on perceived truth has been a subject of experimental research in cognitive and social psychology for nearly five decades, and numerous studies speak to the robustness and pervasiveness of the effect (Dechêne et al., 2010; Henderson et al., 2022).

Although relying on repetition as a cue for truth may generally be a reasonable heuristic, as true information is typically encountered more often than any of its infinite false counterparts, it may be maladaptive in unreliable environments (Orchinik et al., 2025). For example, on social media, algorithms determine the information that we are exposed to based on our previous engagement with content on these and similar channels (e.g., Fanny and Istiono, 2022). This setting may result in a high prevalence of repeated exposure to the same kind of information. Thus, the targeted dissemination of information on these channels has the potential to easily influence public opinion, impact decision-making, and benefit the spread of misinformation (e.g., Pillai and Fazio, 2021).

Considering the practical relevance of the truth effect, developing precise psychological theories about the mechanisms through which repetition affects our beliefs is crucial. A cornerstone of advancing and critically testing theories is by assessing individual differences (Underwood, 1975). However, research on individual differences in the truth effect has yielded ambiguous results, with some studies reporting correlations between individual truth effects and personality variables (e.g., Brashier et al., 2017; Newman et al., 2020; Stump et al., 2022) that other studies failed to detect (e.g., Arkes et al., 1991; Boehm, 1994; De Keersmaecker et al., 2020; Parks and Toth, 2006).

Herein, we address an issue that has been surprisingly underappreciated in previous research, namely, the psychometric properties of truth-effect experiments. As is well known, if measures have low reliability, correlations with these measures are attenuated (Spearman, 1904). Therefore, in individual-differences research, reliability is routinely calculated and reported for standardized instruments such as, for example, personality inventories or cognitive-abilities tests. In the context of behavioral experiments, in contrast, the concept of reliability has limited appeal because experiments are not standardized instruments (Rouder and Haaf, 2019; Rouder and Mehrvarz, 2024). Truth-effect experiments are no exception, as they vary considerably with respect to stimulus material, instructions, and number of replicate trials – all of which affect the reliability of these experiments as a measurement instrument for individual truth effects.

As a first step toward resolving the ambiguity of previous empirical evidence and to put research on individual differences in the truth effect on more solid ground, we investigate how design characteristics of truth-effect experiments affect their psychometric properties. Specifically, in a systematic reanalysis of published and unpublished studies, we analyze how a recently proposed, foundational measure of psychometric goodness – the signal-to-noise ratio γ (Rouder et al., 2023; Rouder and Mehrvarz, 2024) – varies across different design choices. Our aim is to derive recommendations for designing truth-effect experiments that investigate individual differences such that their psychometric properties afford the necessary precision to locate individual truth effects and correlations with other variables.

## The truth effect

Repeated exposure to information is likely to increase the subjective belief that the information is true. This phenomenon has been known and leveraged for centuries, for example, in politics or advertising, to convince other people of certain propositions (e.g., Le Bon, 1895). The effect of repetition on the perceived truth of information is also a well-documented phenomenon in experimental psychology. In a landmark study, Hasher et al. (1977) had participants rate the truthfulness of trivia statements in multiple sessions. Importantly, some of these statements occurred repeatedly across sessions, whereas others occurred only once. These authors found that, independent of the statements’ actual veracity, repeated statements received higher truth ratings than novel ones.

Since this first experimental demonstration of the *truth effect*, it has been replicated numerous times and shown to be remarkably robust across various experimental designs (Dechêne et al., 2010; Henderson et al., 2022). For example, it holds for trivia statements (Fazio et al., 2015), rumors (DiFonzo et al., 2016), opinions (Riesthuis and Woods, 2024), morality judgments (Pillai et al., 2023), fake news (Ecker et al., 2022; Pennycook et al., 2018), product claims (Parks and Toth, 2006), and health-related information (Vellani et al., 2023). Individuals are susceptible to the truth effect throughout their lives, from early childhood (Fazio and Sherry, 2020), through college (Fazio et al., 2015), up to old age (Brashier et al., 2017). It has been demonstrated in the lab and in the field (Fazio et al., 2022), and although warnings about the effect can mitigate it, they do not eliminate it (Nadarevic and Aßfalg 2017). Not even professional fact checkers are immune to the increase of subjective beliefs evoked by repetition (Lin et al., 2024). Overall, the truth effect is a strikingly robust cognitive phenomenon, even though much research has attempted to challenge its limits.

Even though details of the experimental design vary considerably between these studies, all of them follow the same, basic task structure that comprises three phases (e.g., Dechêne et al., 2010; Henderson et al., 2022): In Phase 1, the *exposure phase*, participants are presented with various statements and asked to rate each for interest, sort them into semantic categories, or simply read them. The exposure phase is followed by Phase 2, the *retention phase*, which can last anywhere from a few minutes, during which participants complete a short, unrelated filler task, to several days or weeks. Finally, Phase 3 is the *judgment phase* where participants judge the truth of statements. Critically, half of the statements presented in Phase 3 are repeated from the exposure phase (i.e., old statements), and half of them are new. The truth effect, that is, the increase in perceived truthfulness of information due to mere repetition, is then measured as the difference in truth ratings between repeated and novel statements. At the mean level across individuals, this effect has been shown to be quite stable and medium in size, that is, Cohen’s d≈0.50 (Dechêne et al., 2010). In contrast, the role of individual differences in the truth effect has received notably less attention (e.g., De Keersmaecker et al., 2020; Schnuerch et al., 2021).

### Individual differences in the truth effect

Individual difference research is concerned with the variability of psychological phenomena across individuals, as well as the covariation between phenomena and dispositional variables. This perspective is valuable not only from an idiographic standpoint but also serves as a touchstone for psychological theories (Underwood, 1975). Examining how individual differences in the truth effect relate to other psychological constructs such as personality traits or cognitive abilities enables researchers to test assumptions about underlying mechanisms critically. Identifying these mechanisms, in turn, lays the foundation for the development of precise theory and, ultimately, for designing targeted interventions to mitigate the effect (e.g., Underwood, 1975).

Despite this importance, only a surprisingly small number of studies on individual differences in the truth effect have been published so far (see De Keersmaecker et al., 2020; Schnuerch et al., 2021). Moreover, these studies paint a rather incoherent picture. For example, Brashier et al. (2017) report age-related differences in the truth effect that depend on statement familiarity: Older adults show a reduced truth effect for known falsehoods, consistent with greater reliance on prior knowledge, whereas truth effects for unknown falsehoods were comparable across age groups. In contrast, Parks and Toth (2006) find no association between age and the truth effect for advertising claims. In a series of large-sample studies, De Keersmaecker et al. (2020) assess multiple cognitive-motivational traits (cognitive abilities, preference for deliberation vs. intuition, and need for cognitive closure), and find that none of them moderated the truth effect. Stump et al. (2022), in contrast, find that preference for deliberation and need for cognitive closure moderated the truth effect. These moderation effects were more pronounced after a short retention interval (10 minutes) and decreased after a longer delay (1 week). Based on these results, the authors conclude that the influence of individual differences on the truth effect may depend on retention interval length, possibly due to differences in memory strength. Finally, there is inconclusive evidence regarding need for cognition: Both Arkes et al. (1991) and Boehm (1994) tested the hypothesis that higher need for cognition is associated with larger truth effects, but neither study finds support for this idea. In contrast, Newman et al. (2020) report a positive correlation in one experiment, whereas a subsequent experiment fails to replicate the finding. In a mini meta-analysis of their experiments, these authors found that the moderating effect of need for cognition emerged only in the absence of warnings about false information, pointing to a role of task context rather than stable trait effects. In sum, research on individual differences in the truth effect has produced mixed and sometimes contradictory results, offering little ground for the development of precise psychological theory.

### Reasons for ambiguity

How can we make sense of this ambiguity? One possible explanation is *substantive*: The numerous null findings may reflect the absence of a true common source of variation between the truth effect and the studied personality traits. Although these traits have been found to influence judgment and decision-making in various other domains, the truth effect might be an independent phenomenon. However, this explanation is ultimately unconvincing. After all, some studies do report significant correlations with personality traits, suggesting that *methodological* factors may better explain the inconsistency in findings.

Specifically, the primary methodological concern involves a key statistical concept from classical test theory: reliability. Investigating individual differences with experimental tasks typically relies on correlations between experimental scores and external variables. While it is common knowledge that unreliable measures attenuate correlations (Spearman, 1904), the role of reliability in experimental tasks is often overlooked (Rouder et al., 2026). When examined, however, many such tasks exhibit alarmingly low reliability (Haaf et al., 2025). And under such conditions, localizing true correlations becomes virtually impossible (Rouder et al., 2023).

With these methodological considerations as background, we argue that conflicting evidence regarding correlations between the truth effect and personality traits may reflect variability in measurement reliability. Some studies may have achieved sufficient reliability to localize true or only mildly attenuated correlations, while others may not, resulting in severely attenuated estimates. Thus, before interpreting any pattern of correlations and drawing substantive conclusions about individual differences in the truth effect, we must first ensure that our experimental task affords the necessary psychometric goodness to measure individual effects.

## Psychometrics of individual differences in experimental tasks

Evaluating the psychometric quality of experimental tasks poses a unique challenge (Green et al., 2016; Rouder et al., 2023). Unlike traditional psychometric instruments such as personality inventories or cognitive-abilities tests, experimental paradigms are not fixed, standardized measures. Instead, they are typically tailored to specific research questions and vary along multiple design dimensions – such as instructions, stimulus material, or retention intervals, to name a few. In this sense, there is no “the” truth-effect experiment. Rather, there are numerous variants built upon a common underlying paradigm.

Across all these variants, the critical measure is the difference in truth ratings between repeated and novel statements. This difference score serves as an individual-level estimate of the truth effect, and it is aggregated across multiple trials from both conditions. Consequently, its reliability is inherently linked to the number of replicate trials per person and condition, that is, the *trial size*. Larger trial sizes yield more reliable estimates (Rouder et al., 2026). As a result, any empirical assessment of test–retest reliability for a given experimental design necessarily reflects the influence of trial size.

Of course, trial size is not the only factor affecting the psychometric quality of an experimental task. Other design features – such as the stimulus material, the length of the retention interval, or the choice of instructions – also play a significant role (Green et al., 2016). To isolate the influence of these features and disentangle their contributions from the effects of trial size, we require a metric that is invariant to the number of trials.

Recent advances by Rouder et al. (2023, 2026) and Rouder and Mehrvarz (2024) offer a solution: Drawing on hierarchical modeling, these authors propose a framework that clarifies the relationship between reliability and trial size. From this framework, they derive a foundational metric of psychometric goodness that is independent of trial size, namely, the signal-to-noise ratio γ. In the following sections, we summarize this framework in the context of truth-effect experiments and highlight the limitations of reliability as a measure of psychometric goodness, the advantages of γ, and the role of sample size. Readers interested in the formal underpinnings of this development are referred to the original articles (Rouder et al., 2023; Rouder and Mehrvarz, 2024; Rouder et al., 2026).

### Reliability and trial size

To understand the relationship between reliability and trial size, a formal model is helpful. Consider a standard truth-effect experiment. Each individual *i* provides *L* truth judgments for novel and repeated statements, respectively. Each trial is denoted as $Y_{ij\ell }$, where *i* denotes the individual, i=1,...,I, *j* denotes the condition, j=1,2 for novel versus repeated statements, respectively, and ℓ denotes the replicates in each condition, ℓ=1,...,L. We place the following linear model on $Y_{ij\ell }$ (Schnuerch et al., 2021):

1 $$\begin{aligned} Y_{ij\ell } \sim \mathcal {N}(\alpha _i + x_j \theta _i, \sigma ^2_W), \end{aligned}$$

where $\alpha _i$ is the *i*th individual’s average truth judgment, $x_j = -0.5, 0.5$ is the contrast-coded repetition condition, and $\theta _i$ is the *i*th individual’s true effect of repetition. The component $\sigma ^2_W$ reflects the unsystematic trial-by-trial variability or trial noise within each individual (*W* being short for *within*). The main parameters of interest are the individual truth effects, $\theta _i$. To complete the hierarchical model specification, we place a random-effects model on these parameters:

$$\begin{aligned} \theta _i \sim \mathcal {N}(\nu , \sigma ^2_B), \end{aligned}$$

where ν is the average truth effect in the population and $\sigma _B$ the systematic variability between individuals (*B* being short for *between*).

**Fig. 1:**
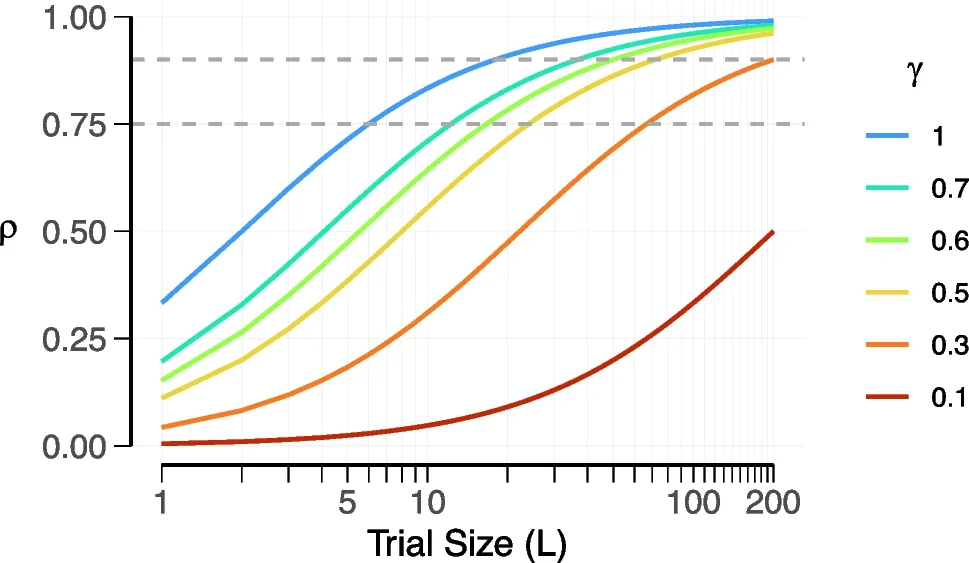
Reliability, signal-to-noise ratio, and trial size. Reliability (ρ) is shown as a function of several signal-to-noise ratios (γ) and number of replicate trials per individual per condition (*L*). The *dashed lines* mark reliability of .75 and .90. Reliability always increases with trial size, but the rate of increase depends on the signal-to-noise ratio: Higher γ values result in faster gains in reliability with additional trials

From the above model follows that the variance of the *observed* individual truth effects $T_i = \bar{Y}_{i2} - \bar{Y}_{i1}$ reflects both systematic variability of true effects between individuals, $\sigma _B^2$, and unsystematic trial noise within individuals, $\sigma _W^2$. Reliability ρ, in turn, defined as the correlation between two hypothetical repetitions of the same experiment with the same individuals, is given by (Rouder et al., 2023):

2 $$\begin{aligned} \rho = \frac{\sigma _B^2}{\sigma _B^2 + 2 \sigma _W^2 / L}. \end{aligned}$$

As evident by this equation, reliability depends on trial size. As *L* increases, so does ρ. Consequently, with an infinite number of trials, we could always achieve perfect reliability. Because it is so dependent on trial size, reliability is an inadequate metric to characterize the psychometric goodness of an experimental task (Rouder et al., 2026).

### Psychometric goodness as signal-to-noise ratio

To capture the psychometric goodness of a task independent of trial size, we must consider the parts of reliability that depend on variability (Rouder and Mehrvarz, 2024; Rouder et al., 2026). By substituting

$$\begin{aligned} \gamma ^2 = \frac{\sigma _B^2}{\sigma _W^2}, \end{aligned}$$

Equation 2 yields

3 $$\begin{aligned} \rho = \frac{\gamma ^2}{\gamma ^2 + 2/L}. \end{aligned}$$

The coefficient $\gamma = \sigma _B/\sigma _W$ does not depend on *L* and it is a *signal-to-noise* ratio. It quantifies the magnitude of true individual differences (the *signal*) relative to trial-by-trial variability or measurement error (the *noise*). As such, γ reflects the inherent ability of a task to measure individual differences independent of trial size, thereby providing a foundational metric for the task’s psychometric goodness (Rouder and Mehrvarz, 2024).

Figure 1 illustrates the relationship between reliability, γ, and trial size. The different curves may reflect different hypothetical truth-effect experiments. As noted above, as trial size increases, reliability increases as well, and this is true for every experiment. What sets them apart, however, is *how fast* reliability increases, that is, the slope of the curves. This slope, the rate of reliability increase per added trial, depends on γ. The higher the signal-to-noise ratio, the fewer trials per person and condition are needed to achieve high reliability.

To summarize, unless one is willing to run hundreds or even thousands of trials per participant, high reliability requires a high signal-to-noise ratio. The coefficient γ quantifies this ratio and thus serves as a metric of psychometric goodness of an experimental task that is independent of trial size. Therefore, for a systematic evaluation of truth-effect experiments, we must investigate how γ varies across different design choices.

### The role of sample size

Having established the importance of trial size and signal-to-noise ratios in experimental studies, we now turn to another quantity frequently emphasized in research on individual differences: the number of individuals in the study, that is, the *sample size*. The focus on sample sizes is natural as small sample sizes add uncertainty to the results. With larger sample sizes, confidence intervals are more narrow, and, consequently, researchers have more confidence in their results.

Intuitively, one might think that we should therefore have more trust in correlation estimates from studies with larger sample sizes. However, whereas the sample size indeed influences the *certainty* of the estimate, the *accuracy* depends on the trial size and the signal-to-noise ratio (Rouder et al., 2023, 2026). This leads to a paradox when large sample sizes are combined with small trial sizes: A small trial size induces systematic bias through attenuation. A large sample size simultaneously leads to narrower confidence intervals around the attenuated estimate. Consequently, with low trial size and increasing sample size, confidence intervals become less likely to cover the true value (Rouder et al., 2026). In other words, not only do large samples not mitigate bias resulting from small trial sizes, they even boost our confidence in the biased estimate (Rouder et al., 2023, 2026).

**Fig. 2:**
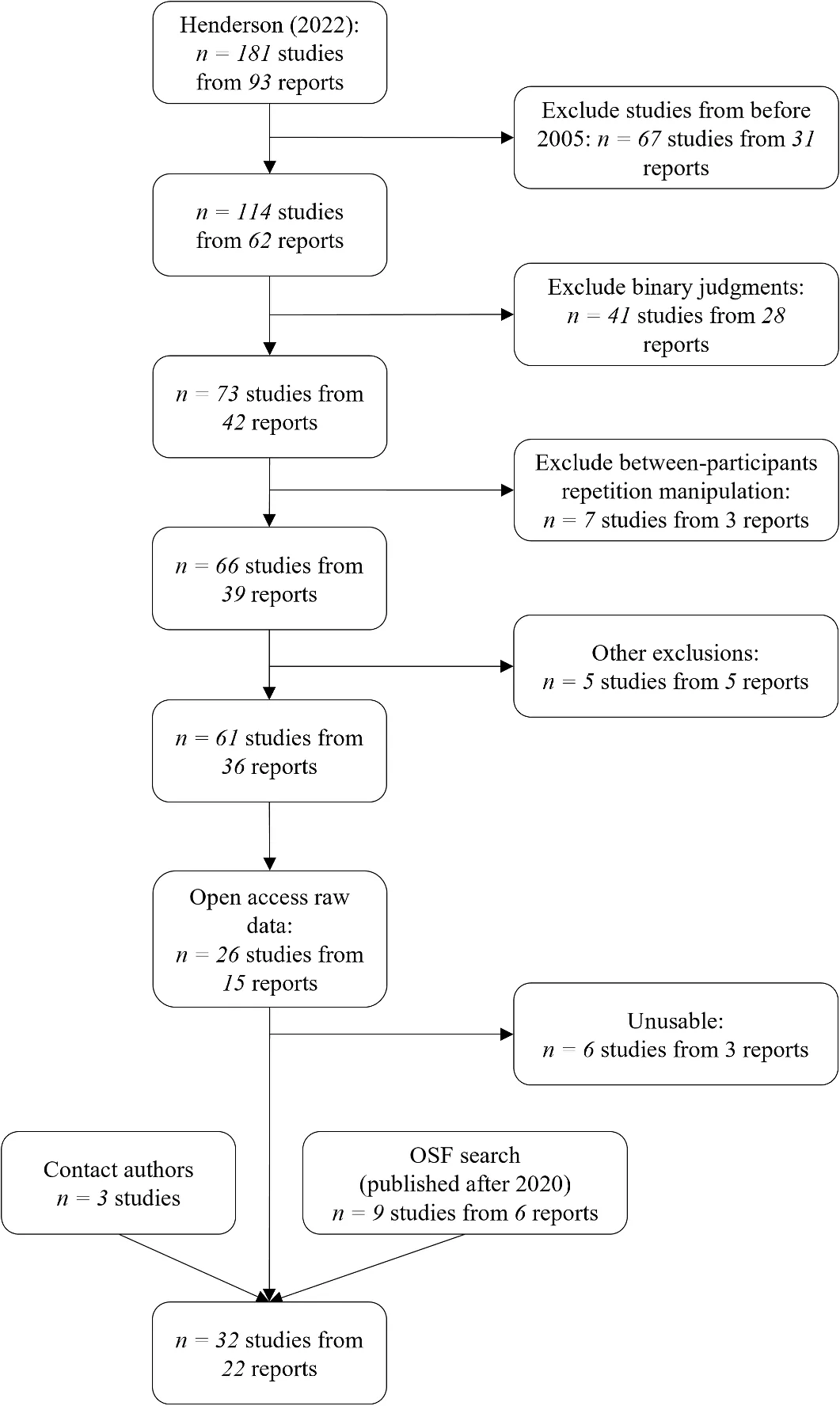
Study selection. The figure outlines the steps of the data selection process. Studies conducted before 2005 were excluded due to limited data availability. Studies using binary judgments were excluded because such responses are incompatible with the linear model used to estimate signal-to-noise ratios. Open access data were deemed unusable if only aggregated data were available, if variable coding was unclear, or if no codebook was provided. In the final step, 15 authors were contacted, and three provided one dataset each

## The present research

In this research, we investigate whether truth-effect experiments afford the necessary psychometric goodness to measure individual truth effects and localize correlations with personality traits and other psychological constructs. Merely increasing trial sizes to ensure sufficient reliability is not a practical approach, as resources of both researchers and study participants are limited. Thus, a good signal-to-noise ratio is important, and it is the deciding measure to characterize the psychometric goodness of an experimental task.

Herein, we present a structured, large-scale analysis of the signal-to-noise ratio γ in published and unpublished truth-effect datasets. We provide a comprehensive overview of general psychometric goodness across studies, and then show how it is influenced by different design choices such as the task administered during Phase 1, the length of the retention interval, the sample population, or the stimulus material. Our results suggest that the ambiguous findings on individual differences in the truth effect may be due – at least, in part – to methodological issues. Psychometric goodness varies considerably across studies, and while γ was low in some of the analyzed datasets, it was quite large in others. Thus, localizing individual differences in the truth effect and correlations with other variables is – in principle – possible. Based on these results, we derive recommendations for designing truth-effect experiments with sufficient psychometric goodness. Although these recommendations are based on retrospective analysis and only descriptive, they provide a valuable basis for future research on individual differences in the truth effect.

**Fig. 3:**
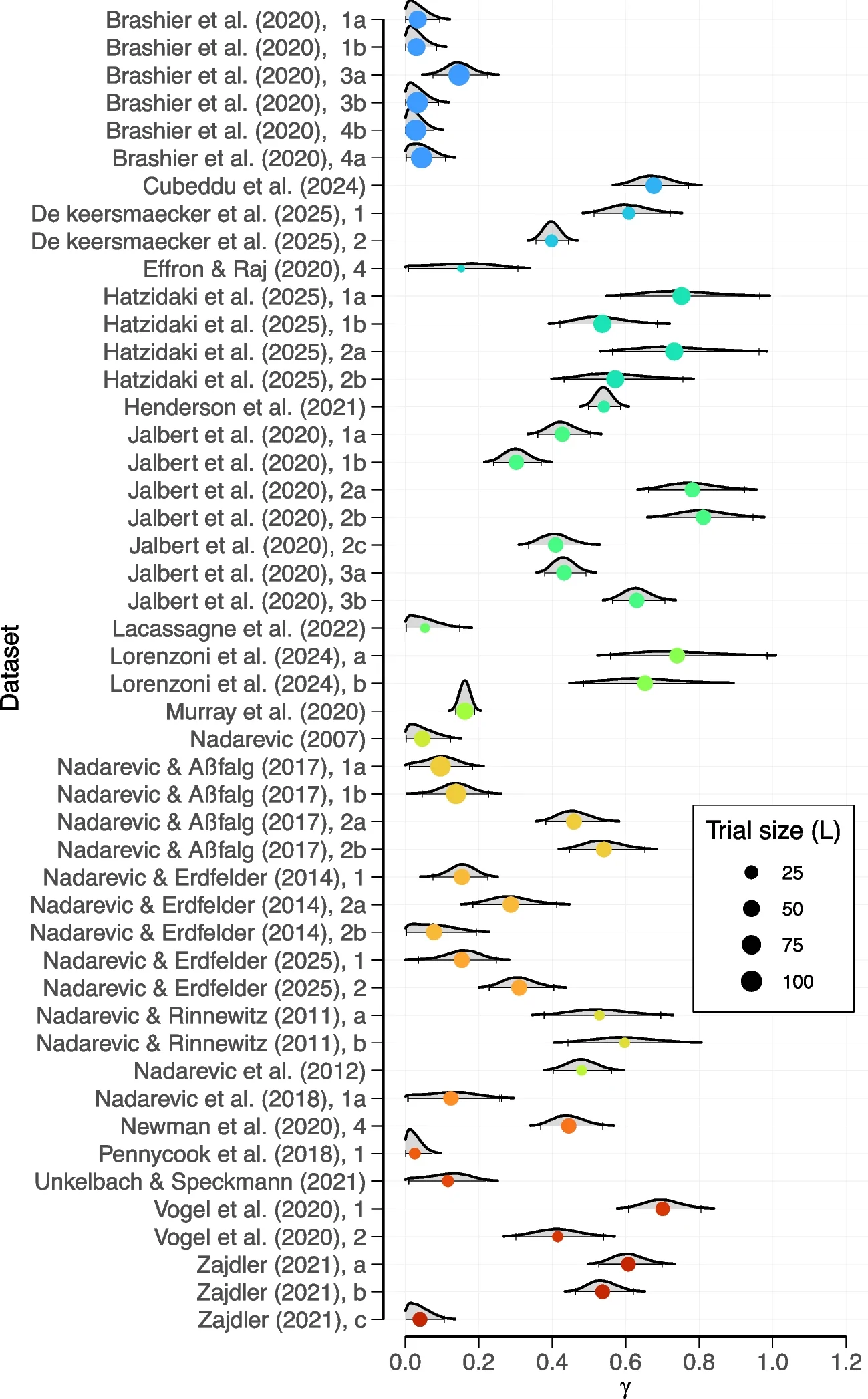
Signal-to-noise ratios for different truth-effect datasets. The signal-to-noise ratio γ and its posterior distribution are plotted for different truth-effect experiments. The 95%-credibility interval is shown through the *error bars*. The point size reflects the trial size *L* and the colors denote the report the study is taken from (i.e., same color indicates the same report). Datasets marked with a *letter* are subsets of the study data that were split due to relevant experimental manipulations

## Method

### Data selection

To identify suitable studies for our analysis, we used Henderson et al. ’s (2022) “systematic and reproducible map of research on the truth effect” as a starting point. Initially intending to conduct a meta-analysis, these authors created a comprehensive database listing 181 truth-effect studies from 93 published and unpublished reports. The map is searchable and includes detailed information on study characteristics and sources.

Starting off with the full map, we first excluded studies published prior to 2005 as the likelihood of retrieving usable datasets among these studies was low due to outdated data formats and inactive author contact information. This criterion led to the exclusion of 67 studies from 31 reports. Next, we excluded 40 studies from 27 reports that used binary (i.e., “true”/“false”) judgments because the linear model development on which the signal-to-noise ratio concept is based does not apply here. We further excluded seven studies from three reports in which repetition was manipulated between participants. An additional five reports were excluded for the following reasons: one was a meta-analysis, one was a reanalysis, one involved a pathological sample, one explicitly labeled the statements as true or false, and one was a pilot study. Of the remaining 62 studies from 36 reports, 27 studies from 15 reports provided open access data. However, six of these studies had to be excluded because either only aggregated data were available (i.e., without trial-level information), variable coding was unclear, or no codebook was available.

Furthermore, we reached out to 15 authors of eligible studies to request access to raw data from their research on the truth effect, where possible. Following this request, we received three additional datasets corresponding to three separate reports. In April 2025, we then systematically assessed further data availability through open access repositories such as the Open Science Framework. To complement the selection of studies listed in the map by Henderson et al. (2022), we focused our search on studies from 2020 onward, as these were not included in the map. Through this search, we identified nine additional studies from six reports. Our final selection of studies thus includes 32 studies from 22 reports, all published after 2005 with either openly accessible or author-provided data. A reproducible record of our study selection process is available on the Open Science Framework (https://osf.io/me367). Figure 2 is a visual summary of the steps outlined above. A list of all datasets and the sources we obtained them through is provided in the Appendix.

For our analyses, some datasets were further split into subsets by between-subjects conditions. These cases included studies in which, for example, some participants received a warning while others did not (Nadarevic and Aßfalg, 2017), or where different tasks were administered during Phase 1 (Brashier et al., 2020; Zajdler, 2021). In Fig. 3, these subsets that represent different conditions from the same experiment are marked with letters. For studies with multiple measurement occasions involving the same participants, only the first measurement occasion is included in the analysis unless otherwise specified. More detailed information on the datasets is provided in the supplement on the Open Science Framework (https://osf.io/me367).

### Analysis

Before analysis, we rescaled all truth judgments to range from -1 to 1 for the sake of comparability between studies. Let *Y* denote the truth judgment on the original rating scale. The rescaled truth judgment $Y^{*}$ is then

$$\begin{aligned} Y^{*} = \frac{Y - Y_{\text {mid}}}{Y_{\max } - Y_{\text {mid}}}, \end{aligned}$$

where $Y_{\text {mid}}$ denotes the midpoint of the respective scale, $Y_{\text {mid}} = (Y_{\min } + Y_{\max })/2$. $Y_{\min }$ and $Y_{\max }$ denote the minimum and maximum, that is, the “definitely false” and “definitely true” endpoints of the scale, respectively. Individual effects can then range from -2 if all repeated statements are rated as definitely false and all novel statements as definitely true, to 2 if all repeated statements are rated as definitely true and all novel statements as definitely false.

We calculated the trial size as the average number of truth judgments that were given per condition across participants in one experiment. We used the average number of truth judgments, as some experiments had timed judgments, resulting in divergent numbers of responses per participant within the same experiment. Trials without response were excluded prior to averaging.

We analyzed the rescaled truth judgments with a Bayesian hierarchical linear model based on the trial-level model given by Eq. 1. The complete model specification, including all prior settings, is provided in the appendix. We ran the model using Markov chain Monte Carlo (MCMC) sampling with brms (Bürkner, 2017) in R (R Core Team, 2025). For each dataset, 8 chains were run with 2, 000 iterations per chain, resulting in 16, 000 posterior samples in total. From the posterior samples for $\sigma _B$ and $\sigma _W$, we then calculated posterior samples for the signal-to-noise ratio γ. Unlike the method-of-moments estimator proposed by Rouder and Mehrvarz (2024), this MCMC-based approach allows us to quantify uncertainty in the γ estimate because we obtain not only a point estimate but the entire posterior distribution. R code to reproduce all analyses, figures, and tables reported here may be retrieved from the Open Science Framework (https://osf.io/me367).

## Results

Figure 3 presents the posterior distributions of the signal-to-noise ratio γ for each study, along with posterior means and 95% credibility intervals. Overall, there is substantial variation in γ across studies, ranging from as low as 0.03 – indicating that the trial-level standard deviation is more than thirty times greater than the between-person standard deviation – to as high as 0.81, where systematic variability is nearly as large as the noise. This wide range implies that the psychometric quality of truth-effect experiments differs markedly between studies. In cases with low γ, several hundred trials per participant would be required to achieve acceptable reliability. In contrast, studies with γ values of 0.60 or higher suggest that it is, in principle, possible to localize individual differences in the truth effect even with moderate trial sizes. Across all studies, the median signal-to-noise ratio is γ=0.41. For reference, γ values in widely used experimental tasks such as Stroop or Flanker paradigms are typically much lower, averaging around γ=0.10 (Rouder and Mehrvarz, 2024).

**Fig. 4:**
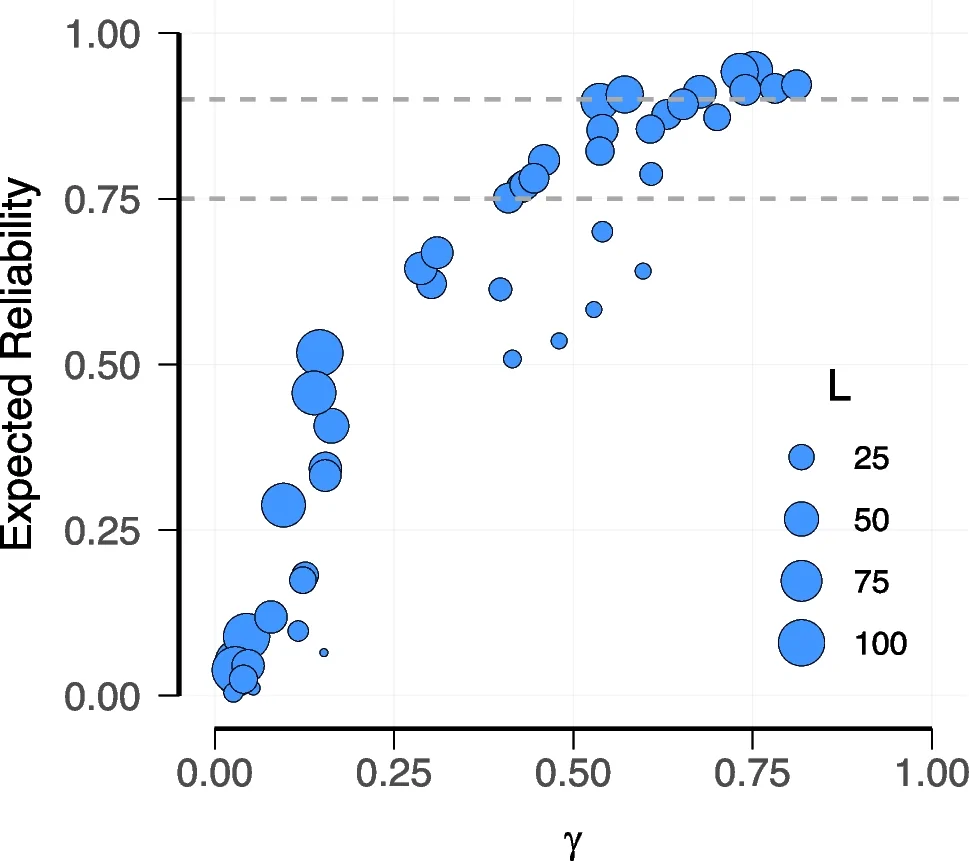
Expected reliability in different truth-effect datasets. Expected reliability (as per Eq. 3), signal-to-noise ratio (γ), and trial size (*L*) are plotted for different truth-effect experiments. The *dashed lines* mark reliability of .75 and .90. While larger trial sizes generally result in higher reliability, the determining factor is the signal-to-noise ratio: When γ is too low, increases in trial size yield minimal improvements in reliability

To clarify the practical implications of these findings, Fig. 4 shows the expected reliability for each study as a function of both γ and trial size *L*. As illustrated, even the largest observed trial sizes (L=100) cannot compensate for low psychometric goodness (i.e., γ<0.40). Conversely, moderately high values of γ (i.e., around 0.50) still yield insufficient reliability when paired with small trial sizes (i.e., L<25). Across all studies, the median expected reliability is .62. These findings emphasize that high reliability requires both adequate psychometric goodness and sufficient trial sizes. Encouragingly, several of the studies analyzed here met both conditions and thus provided the basis to reliably measure individual differences in the truth effect.

**Fig. 5:**
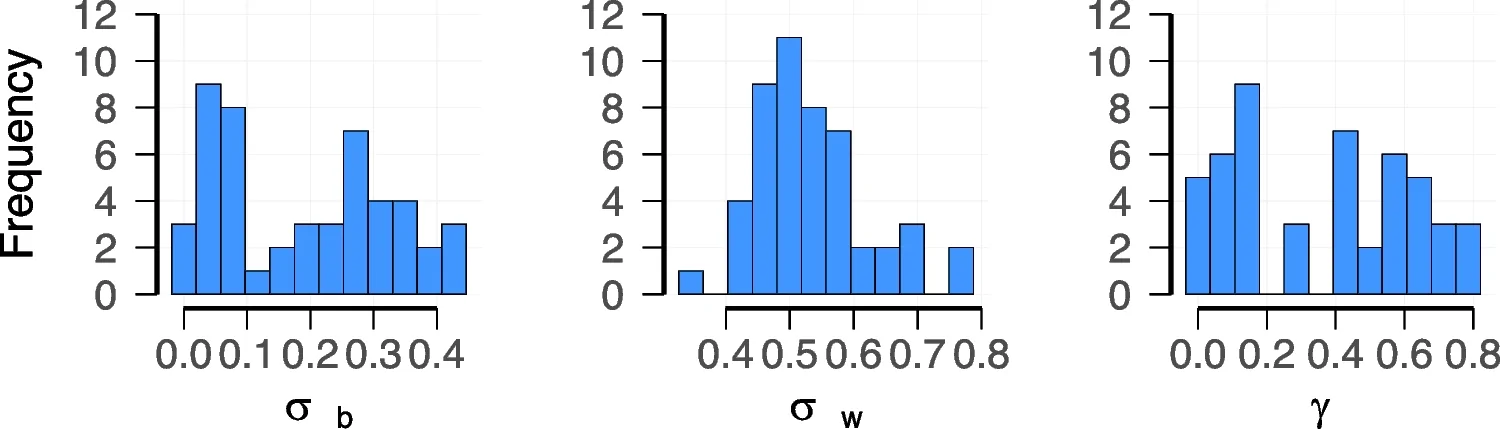
Observed frequencies of $\sigma _B$, $\sigma _W$, and γ. Observed distributions of variability between individuals ($\sigma _B$), trial variability within individuals ($\sigma _W$), and signal-to-noise ratios (γ). Differences in γ are primarily driven by variability in $\sigma _B$, while $\sigma _W$ is more consistent across datasets

What drives the observed variability in psychometric goodness across studies? A higher signal-to-noise ratio γ can result from greater systematic variability between individuals (i.e., a larger $\sigma _B$) or from reduced trial-level noise (i.e., a smaller $\sigma _W$). Both components can be influenced by features of the experimental design or characteristics of the sample. As shown in Fig. 5, we observe variability in both $\sigma _B$ and $\sigma _W$ across studies. However, while estimates of trial-level noise ($\sigma _W$) are relatively tightly concentrated around 0.50 ($SD_{\sigma _W} = 0.09$), estimates of between-person variance ($\sigma _B$) show greater dispersion ($SD_{\sigma _B} = 0.14$). This asymmetry indicates that differences in γ are primarily driven by variability in the extent to which the experimental design elicits systematic variation in individual effects.

**Fig. 6:**
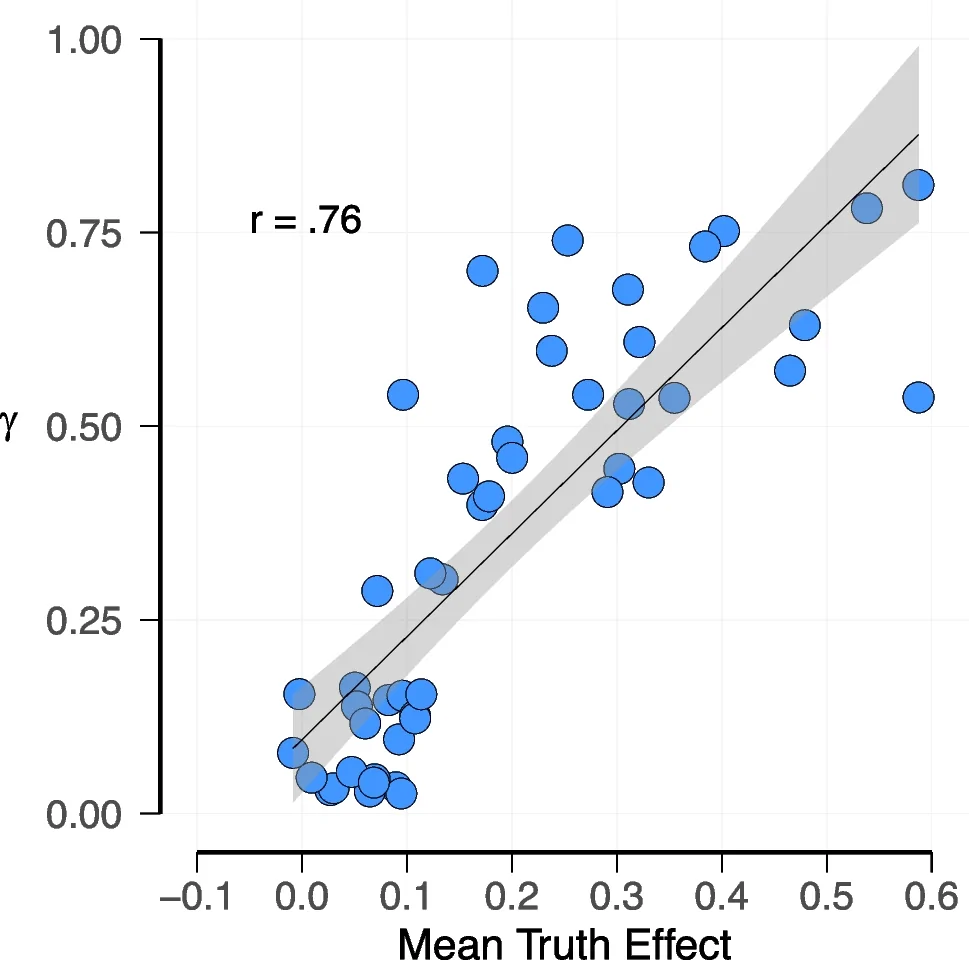
Mean truth-effect size and signal-to-noise ratio. Mean truth-effect size and signal-to-noise ratio (γ) show a strong positive correlation across studies. The *black line* represents the correlation of r=.76, and the *shaded area* depicts the corresponding confidence interval

The strong association between psychometric goodness and the magnitude of the mean truth effect further supports this interpretation. As shown in Fig. 6, the signal-to-noise ratio γ is highly correlated with the average size of the truth effect. This relationship is theoretically expected. Prior work suggests that, in the case of the truth effect, a *dominance assumption* holds: individuals tend to show either a positive effect or no effect, but nobody shows a true negative effect (De Keersmaecker et al., 2026; Schnuerch and Rouder, 2026). Under this assumption, a small mean effect necessarily implies a restricted range of individual differences, since the distribution of individual effects is bounded below by zero (Rouder et al., 2023). In contrast, a larger mean effect permits greater dispersion across individuals, which in turn increases the systematic variance between individuals, and thus, the signal-to-noise ratio γ. Consequently, experimental designs that enhance the average strength of the truth effect also improve the task’s ability to localize individual effects. This finding highlights the dual benefit of systematically assessing and optimizing parameters of the experimental design – not only to maximize the average effect size, but also to enhance the psychometric goodness for measuring individual effects.

In summary, the psychometric goodness of truth-effect paradigms varies substantially across studies. The good news is that high γ values – and, consequently, the reliable detection of individual differences and correlations – are attainable. The key challenge, however, lies in identifying the specific design characteristics that promote high psychometric goodness. In the following, we therefore investigate how γ varies with key experimental characteristics and, based on these findings, derive practical recommendations for designing truth-effect studies that support reliable measurement with feasible trial sizes.

## Experimental characteristics and the signal-to-noise ratio

To identify critical design features, we systematically examined how the signal-to-noise ratio varied across datasets with different initial tasks, stimulus material, samples, study settings, retention intervals, and filler tasks. Although disentangling the individual effects of specific and often highly collinear experimental design choices is not possible in this retrospective analysis, we explore general patterns and associations that offer useful guidance for experimental planning. To critically assess causal relationships, further experimental evidence and testing is necessary. Nevertheless, this research provides a first step for identifying critical design choices when planning experiments for localizing structure in individual differences in the truth effect.

### Initial task

We begin with the choice of task that participants perform during Phase 1, the *initial task*. Typically, participants sort the presented statements into semantic categories, rate them according to their personal interest, or simply read them. However, when initial truth judgments are provided at exposure (Phase 1), the truth effect vanishes at the average level (Brashier et al., 2020; Nadarevic and Erdfelder, 2014). Consequently, following our line of argument presented above, the choice of initial task should influence the signal-to-noise ratio.

**Fig. 7:**
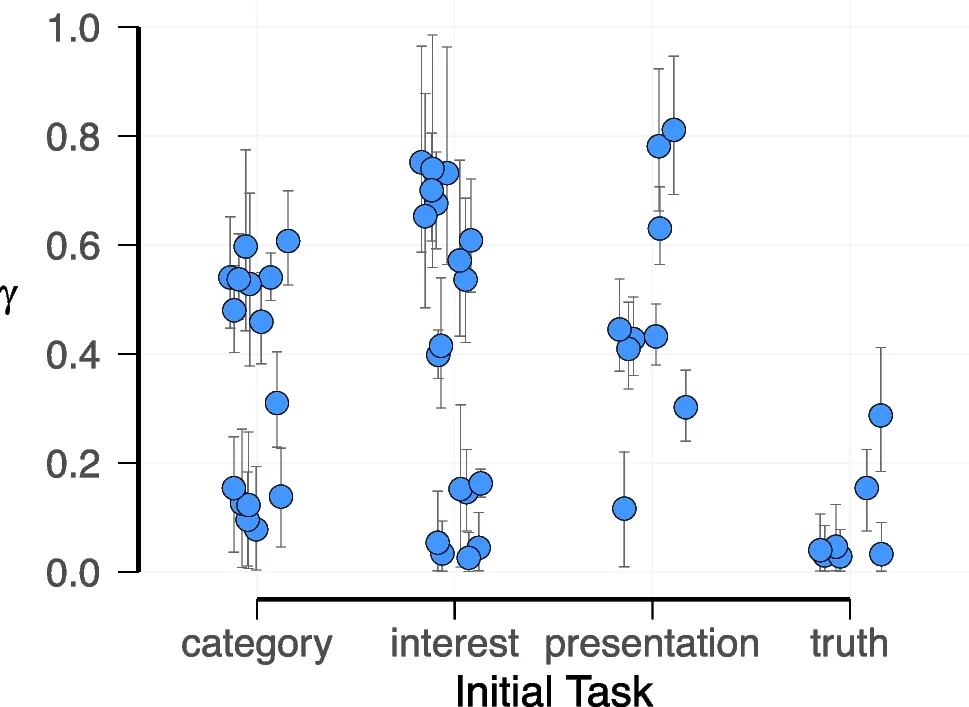
Initial task and signal-to-noise ratio. Signal-to-noise ratios (γ) are displayed by initial task. *Error bars* represent the 95% credible intervals. Signal-to-noise ratios are systematically low when participants perform truth judgments as the initial task

Figure 7 illustrates how γ varies across different initial tasks in the analyzed datasets. While there is not one task that guarantees high psychometric goodness, γ is systematically lower for studies that implement truth judgments during Phase 1 (*Mdn* =0.04 versus *Mdn* =0.44). This is primarily driven by the low variability between individuals ($\sigma _B$) when truth judgments are performed during Phase 1 (*Mdn* =0.02) as opposed to other tasks (*Mdn* =0.25). In contrast, trial variability within individuals (${\sigma _W}$) is relatively consistent for different initial tasks (*Mdn* =0.49 for truth judgments versus *Mdn* =0.52 for other tasks).

As all design choices are strongly intertwined, we singled out one dataset as an exemplary demonstration where everything but the initial task is equal. Zajdler (2021) manipulated Phase 1 to determine whether individual differences in the truth effect could be explained by differences in innate skepticism elicited by different instructions and tasks. Comparing an experimental group where participants performed truth judgments during Phase 1 with a control group where participants sorted statements into knowledge categories, we find that γ is lower in the former, $\gamma _{\text {truth}} = 0.04$, 95% CI [0.002; 0.11], $\gamma _{\text {category}} = 0.61$, 95% CI [0.53; 0.70]. Importantly, this difference is driven by a decrease in the variability between individuals, $\sigma _{B, \text {truth}} = 0.02$, $\sigma _{B, \text {category}} = 0.29$, whereas the trial variability within individuals is unaffected by this design choice, $\sigma _{W, \text {truth}} = 0.49$, $\sigma _{W, \text {category}} = 0.48$.

**Fig. 8:**
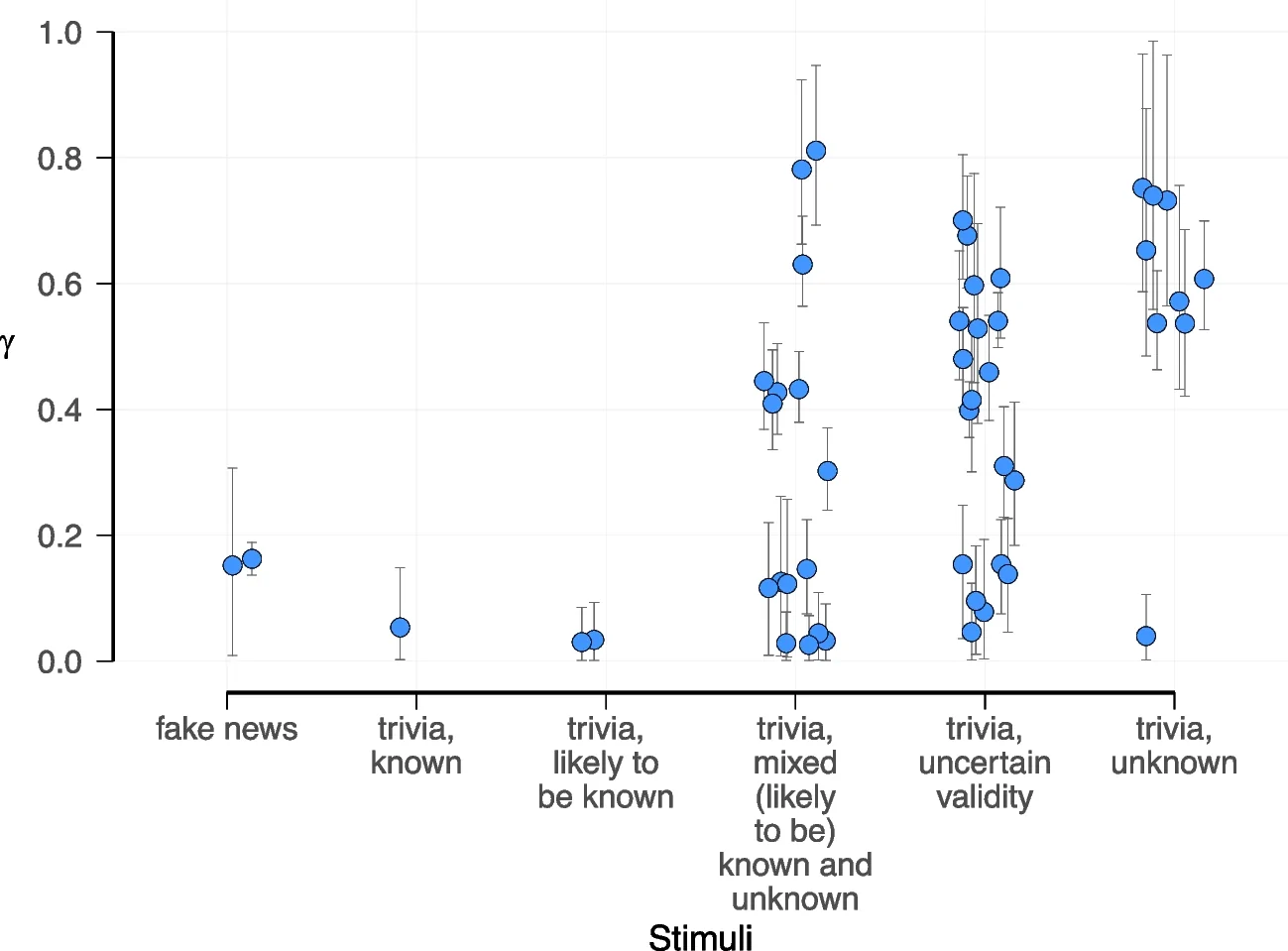
Stimulus material and signal-to-noise ratio. Signal-to-noise ratios (γ) are plotted by stimulus material across studies. *Error bars* represent the 95% credible intervals. Signal-to-noise ratios are highest for unknown trivia statements, with one exception: a subset in which participants performed truth judgments in Phase 1

While the above demonstrates that performing truth judgments during exposure limits psychometric goodness for studying individual differences in the truth effect, prior work suggests that the impact of the exposure phase extends beyond this particular task. Specifically, it is the depth of processing induced during exposure that has been shown to affect the magnitude of the truth effect. For example, Unkelbach and Rom (2017, Experiment 3) demonstrated larger truth effects at the aggregate level following deeper encoding (e.g., self-referential writing task) compared to shallow encoding (e.g., indicating the spatial position of a statement) during exposure. The initial tasks typically used in truth-effect experiments differ substantially in the extent to which they require semantic elaboration of the statements. Simply reading statements affords relatively shallow processing, whereas categorization tasks require participants to process and understand statement content, and interest ratings may further engage self-referential processing by requiring individuals to relate the statements to themselves. From the perspective of individual differences, in turn, deeper processing during exposure may be advantageous because larger effects are associated with greater variability between individuals. Consequently, initial tasks that avoid direct truth judgments but nevertheless encourage deeper processing, such as a categorization task or interest ratings, may be better suited for studies aiming to capture individual differences in the truth effect reliably.

### Stimulus material

The truth effect has been demonstrated across a range of stimulus materials, particularly trivia statements (Henderson et al., 2022) but also fake news headlines and opinion statements. In the analyzed datasets, we identified six categories of stimulus material: known trivia statements (i.e., statements that can be correctly identified as true or false by a large majority of participants), likely to be known trivia statements (i.e., statements that were correctly identified as true or false by around 60% of participants in a norming study), unknown trivia statements (i.e., trivia that was known by less than 1% of participants in a norming study), a mixture of (likely to be) known and unknown trivia statements, trivia statements with uncertain validity (i.e., unknown statements that received medium truth ratings in a pre-study), and fake news headlines. Different effects of the choice of stimulus material on the signal-to-noise ratio are conceivable. For example, using a mix of statements with known and unknown truth status may increase unexplained trial-by-trial variability. Relying exclusively on known trivia statements, in contrast, may constrain variability between individuals as shared prior knowledge may reduce the influence of repetition on truth judgments.

Figure 8 shows how the signal-to-noise ratio varies across different types of stimulus material. We observe the highest γ values for unknown trivia statements – with one exception, namely, the condition from Zajdler (2021) where participants judged the truth of statements in Phase 1. As expected, the variation in γ reflects variation in both $\sigma _B$ and $\sigma _W$. Systematic variability between individuals is largest when unknown trivia statements are used (*Mdn* =0.32), and smallest for known (*Mdn* =0.02) or likely to be known trivia statements (*Mdn* =0.03).

Trial-by-trial variability is affected when the stimuli vary in difficulty within participants. It is highest when trivia statements are likely to be known (*Mdn* =0.79) or consist of a mix of (likely to be) known and unknown statements (*Mdn* =0.59). When only known statements are used, in turn, within-person trial variability is reduced (*Mdn* =0.36). As mentioned above, however, variability between individuals is even lower in these cases, resulting in rather low γ values. Overall, using exclusively unknown trivia statements appears to be the most effective choice of stimulus material for studying individual differences in the truth effect.

Naturally, relying on unknown trivia statements only may not be feasible in the context of applied research questions that specifically investigate the truth effect across different stimulus materials. For example, to test the impact of implausibility on the effect of repetition, Pennycook et al. (2018, Experiment 1) curated an item pool consisting of unknown trivia statements and a few known truths and falsehoods, such as “The earth is a perfect square.” We reanalyzed the signal-to-noise ratio for this dataset, this time using only the unknown trivia statements. Together with our initial analysis, we obtain two comparable conditions where all design choices but the stimulus material are identical. When both known and unknown statements are included, trial noise is high ($\sigma _W = 0.68$), while the variability between individuals is low ($\sigma _B = 0.02$). In contrast, analyzing only unknown trivia statements yields reduced trial variability ($\sigma _W = 0.50$) and increased between-individual variability ($\sigma _B = 0.16$), resulting in a better signal-to-noise ratio. These results suggest that, when the exclusive use of unknown trivia statements is not feasible, psychometric goodness may be improved by selecting stimuli of similar difficulty for analysis. Note, however, that this approach necessarily comes with a reduced trial size, which adversely affects reliability.

A different approach is to reduce trial noise by explicitly modeling the factual truth of statements as an additional predictor. For example, reanalyzing the data from Pennycook et al. (2018, Experiment 1) with factual truth as a predictor, we obtain a lower estimate of trial noise ($\sigma _w = 0.43$) and a higher estimate for the variability between individuals ($\sigma _B = 0.12$). This result suggests that, when some of the statements are known, part of the variance previously attributed to random noise can be more appropriately explained by factual item truth. However, for item sets composed solely of unknown or uncertain trivia statements, which applies to most of the analyzed datasets, including factual truth as a predictor has minimal impact. Therefore, and because information on the items’ factual truth status was not consistently available for all datasets, we included it as a predictor only in this illustrative example.

### Sample and study setting

An important and widely debated design consideration in experimental research concerns the selection of a particular sample. Psychological research relies heavily on convenience samples of college students, a practice that has been criticized due to concerns about the generalizability of findings (e.g., Peterson and Merunka, 2014). College students have been found to differ systematically from general-population samples along multiple dimensions, including task-related behaviors (e.g., time spent on tasks, attentional engagement) as well as psychological characteristics such as personality traits, need for cognition, and personal values (e.g., Weigold and Weigold, 2022). These latter factors are particularly critical for research on individual differences as the increased homogeneity in (psychology) student samples may obscure the true variability in the general population and thus attenuate estimates of individual differences.

The selection of a particular sample is closely intertwined with the study setting – specifically, whether an experiment is conducted in a laboratory or online. Lab-based studies typically rely on college student samples, whereas general-population samples are more commonly recruited through online platforms. As a result, online studies are often associated with more heterogeneous samples, potentially increasing between-person variability and, in turn, improving the signal-to-noise ratio. However, online settings may also introduce additional sources of trial-level noise due to factors such as reduced attentional control, environmental distractions, or lower task engagement. These factors could counteract the benefits of increased heterogeneity by elevating within-person variability, thereby diminishing the overall signal-to-noise ratio.

**Fig. 9:**
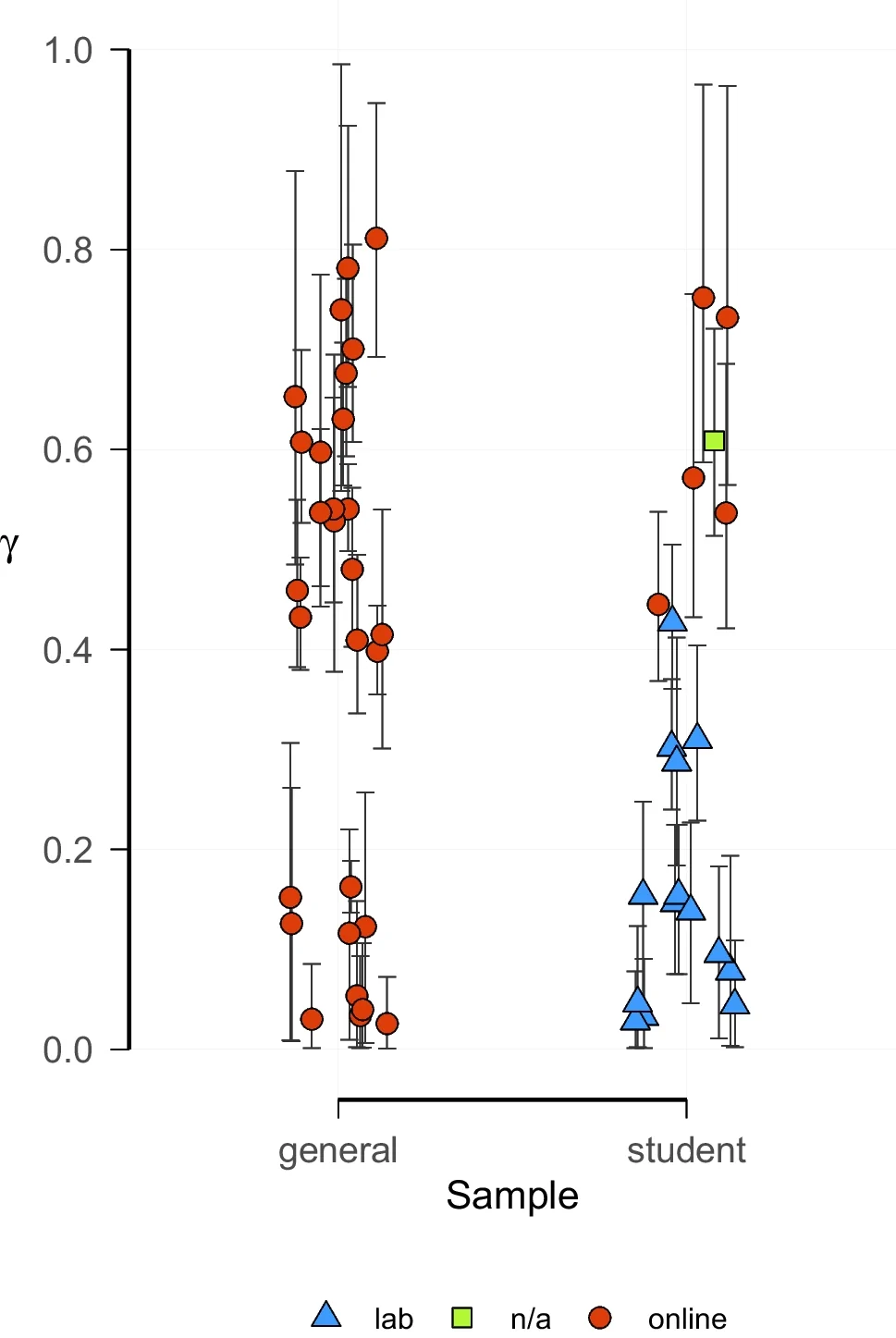
Sample, study setting, and signal-to-noise ratio. Signal-to-noise ratios (γ) are shown by sample and study setting across studies. *Error bars* represent the 95% credible intervals. Signal-to-noise ratios tend to be higher for general samples and for student samples in online settings. Exceptions include studies in which participants completed truth judgments in Phase 1 or that used less suitable stimulus material

Figure 9 displays the relationship between γ, the type of sample, and study setting. Overall, studies using samples from the general population exhibit higher signal-to-noise ratios (*Mdn* =0.46) compared to those using student samples (*Mdn* =0.22). Notable exceptions of general-population samples with particularly low signal-to-noise ratios (bottom left corner of Fig. 9) include studies in which participants performed truth judgments in Phase 1 or that employed less suitable stimulus material (i.e., known or likely to be known trivia statements or fake news headlines). Differences in γ are driven by differences in ${\sigma _B}$ (general samples: *Mdn* =0.25; student samples: *Mdn* =0.11). In contrast, trial-by-trial variability $\sigma _W$ was roughly equal in general (*Mdn* =0.51) and student (*Mdn* =0.55) samples.

Relatedly, online studies exhibit notably larger signal-to-noise ratios than lab-based studies (online: *Mdn* =0.50; lab: *Mdn* =0.14). To disentangle the selective influence of sample and study setting, we can focus on the subgroup of studies that employed student samples. Among these studies, online studies also yield substantially higher γ values (*Mdn* =0.57) than lab-based studies (*Mdn* =0.14). Although trial noise $\sigma _W$ is slightly lower in laboratory (*Mdn* =0.48) compared to online settings (*Mdn* =0.56), the variability between individuals, $\sigma _B$, is also markedly reduced for laboratory (*Mdn* =0.07) compared to online studies (*Mdn* =0.34). As the decrease in between-individual variability outweighs the marginal decrease in trial variability, the net result is a lower signal-to-noise ratio in laboratory studies compared to online studies.

**Fig. 10:**
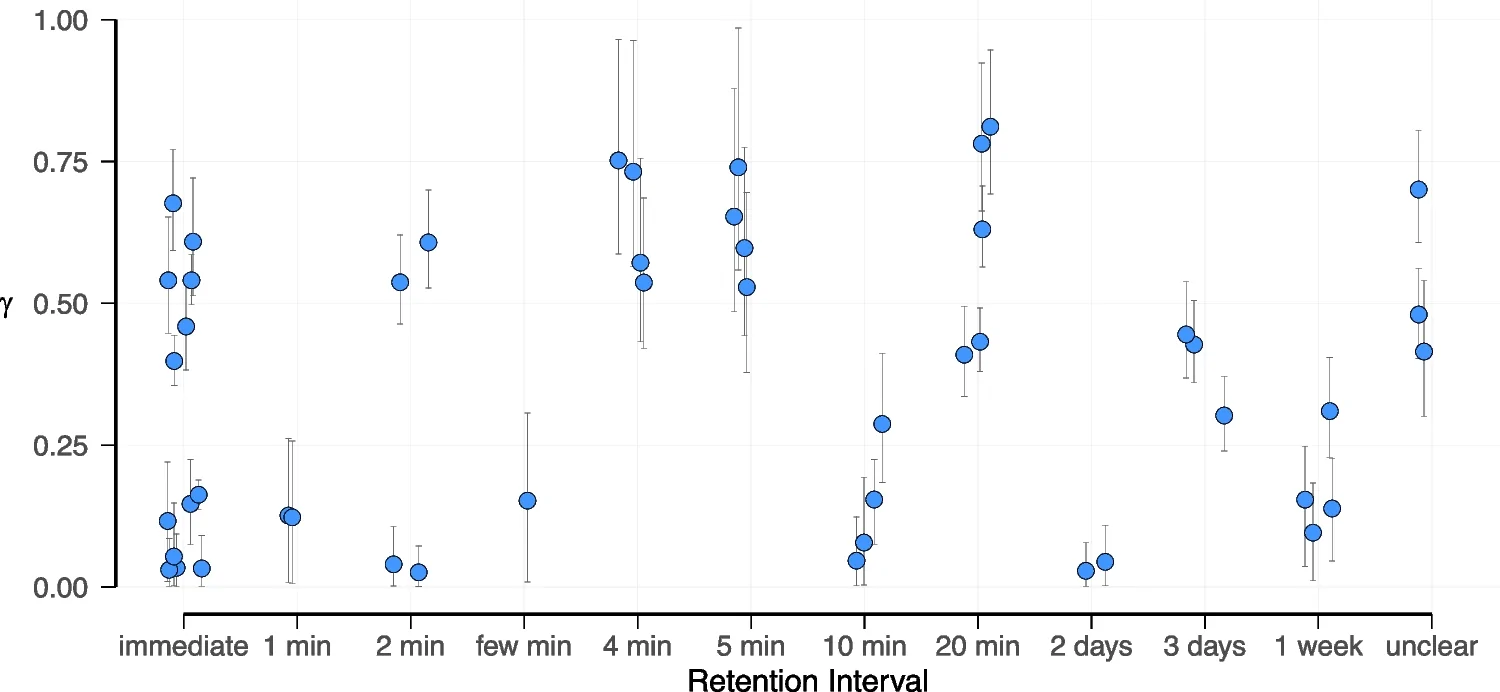
Retention intervals and signal-to-noise ratio. Signal-to-noise ratios (γ) are plotted by retention interval across studies. *Error bars* represent the 95% credible intervals. The relationship between retention interval and signal-to-noise ratios is difficult to systematize

### Retention interval

Another critical design choice in truth-effect experiments is the length of the retention interval in Phase 2. Short retention intervals – administered in a single session together with Phase 1 and 3 – are often preferred for practical reasons, as they do not require follow-up participation. Correspondingly, same-session intervals are the most commonly implemented (but see Henderson et al., 2021; Nadarevic and Erdfelder, 2014; Stump et al., 2022; Stump et al., 2024). Regarding individual differences, previous research indicates that the length of the retention interval affects the variability of individual truth effects. Whereas Schnuerch et al. (2021) report strong evidence for large individual variability in the truth effect under same-session retention intervals (see also Schnuerch and Rouder, 2026), they found support for a lack of individual differences in studies with 1-week retention intervals. Similarly, Stump et al. (2022) report that the truth effect was moderated by preference for deliberation and need for cognitive closure following a short delay of ten minutes. However, these moderation effects decreased after a delay of one week, suggesting that the retention interval modulates the extent to which individual differences shape truth judgments. Taken together, these empirical findings indicate that retention interval length is likely to be another critical design feature that influences signal-to-noise ratios.

Figure 10 shows the observed signal-to-noise ratio across different retention intervals. Note that this analysis only includes studies that did not specifically manipulate the retention interval. The choice of retention interval varies considerably across studies, leaving relatively few data points per specific level. Therefore, it is difficult to draw strong conclusions from these results.

**Fig. 11:**
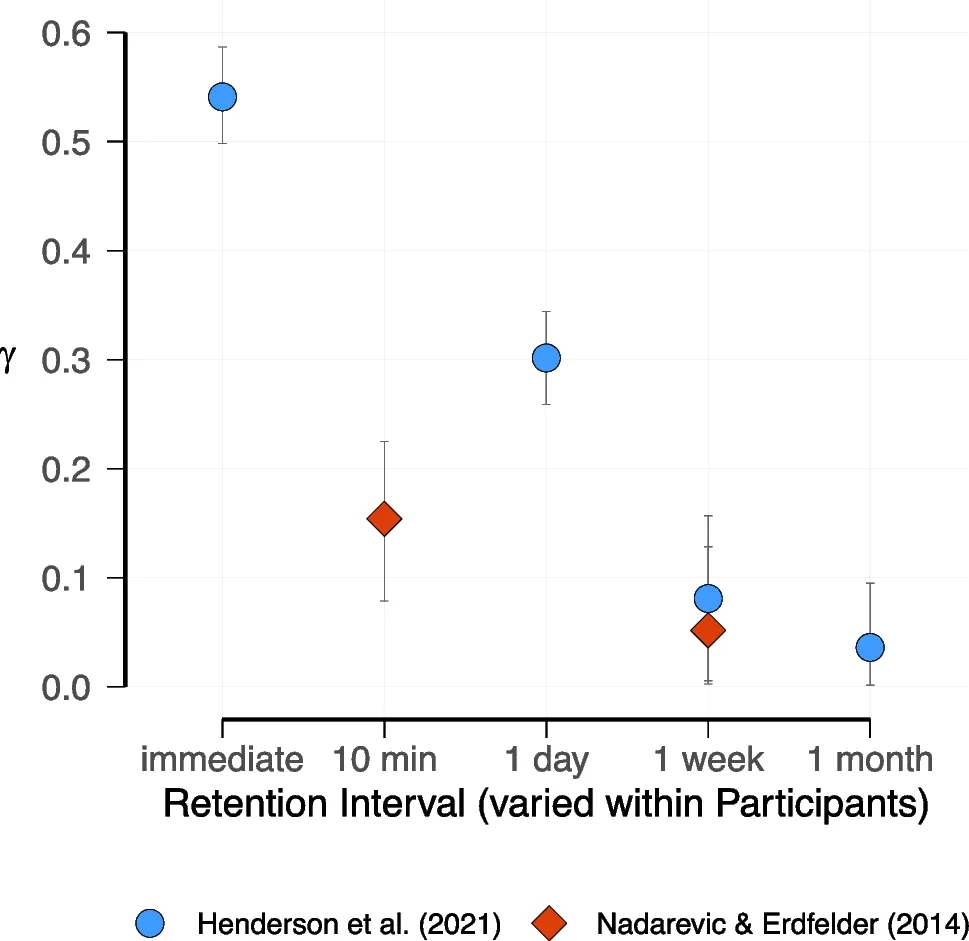
Different retention intervals and signal-to-noise ratio within the same participants. Signal-to-noise ratios (γ) are plotted by retention interval across different sessions with the same participants in two studies. *Error bars* represent the 95% credible intervals. Signal-to-noise ratios decrease with prolonged retention intervals

Instead, we can take a closer look at two studies that manipulated retention interval length within participants. In these studies, participants completed a single exposure phase followed by multiple truth judgment phases administered after different retention intervals. Figure 11 shows how γ varies across the different interval lengths.^1^ The pattern is consistent in both studies: Longer retention intervals are associated with reduced signal-to-noise ratios. As expected, this pattern is due to a decrease in $\sigma _B$ (see Table 1). While trial noise remains relatively constant across retention intervals, the variability between participants decreases as the retention interval increases. Thus, from these data, we conclude that psychometric goodness is highest when the truth effect is assessed after a short, within-session retention interval.

**Table 1:** Retention interval, signal-to-noise ratios, and variability

Dataset	Retention interval	γ	$\sigma _B$	$\sigma _W$
Henderson et al. (2021)	immediate	0.54	0.29	0.53
Henderson et al. (2021)	1 day	0.30	0.16	0.53
Henderson et al. (2021)	1 week	0.08	0.04	0.52
Henderson et al. (2021)	1 month	0.04	0.02	0.52
Nadarevic and Erdfelder (2014)	10 min	0.15	0.07	0.45
Nadarevic and Erdfelder (2014)	1 week	0.05	0.02	0.46

*Note.* Signal-to-noise ratios (γ), variability between individuals ($\sigma _B$), and trial noise ($\sigma _W$) by retention interval length. Signal-to-noise ratios decrease with longer retention intervals, driven by a decrease in $\sigma _B$

### Filler task

Although some truth-effect experiments proceed directly to the judgment phase, studies with same-session retention intervals typically include a filler task between exposure and judgment. As established above, such short intervals are generally advantageous for studying individual differences, but it remains unclear whether and how the characteristics of the task administered to fill the retention interval may influence subsequent truth judgments. In line with this consideration, Henderson et al. (2022) noted a lack of standardization in filler tasks and suggested that, much like the initial task at exposure, they may influence the magnitude of the truth effect. Prior work further supports this notion: High cognitive capacity and motivation *at judgment* reduced the truth effect relative to conditions in which participants lacked motivation, capacity, or both (Garcia-Marques et al., 2016, Experiment 1). Thus, filler tasks may exert similar influences by taxing cognitive resources, interfering with the material, or reducing strategic rehearsal of previously encountered statements, thereby affecting signal-to-noise ratios in same-session truth-effect paradigms.

**Fig. 12:**
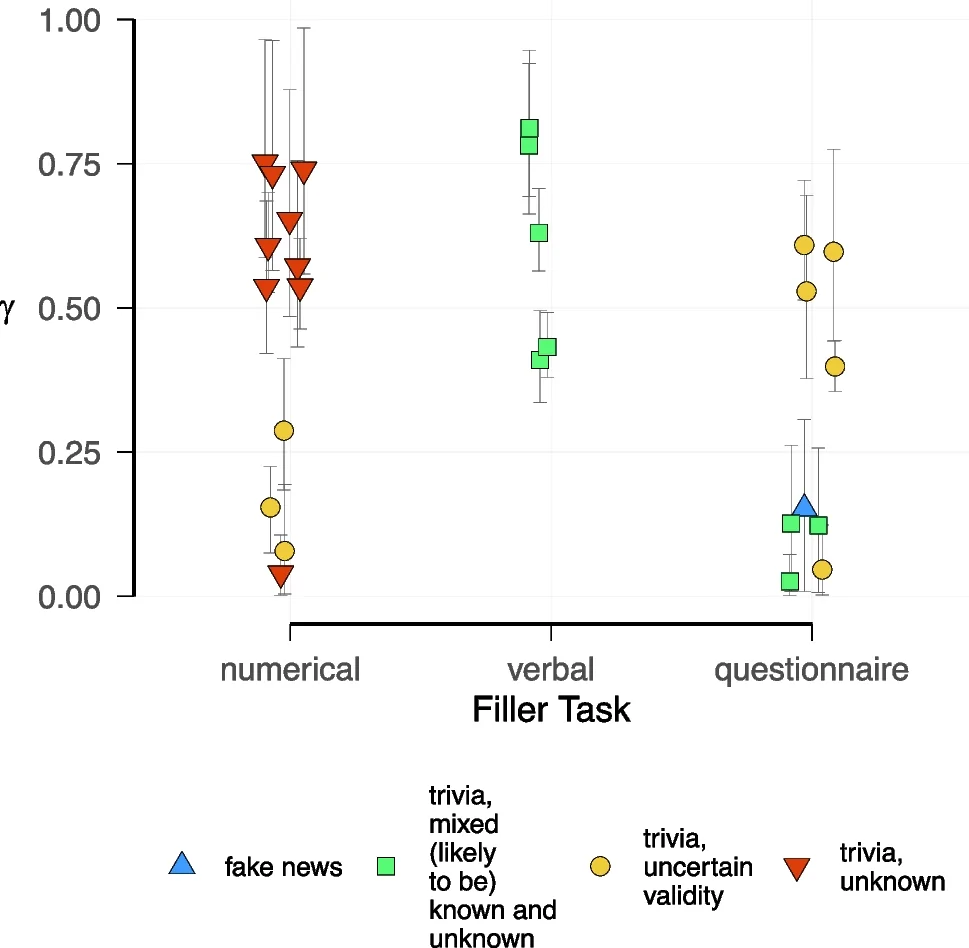
Different filler tasks and signal-to-noise ratio in same-session designs. Signal-to-noise ratios (γ) are plotted by filler task across studies with same-session delays. *Error bars* represent the 95% credible intervals. Questionnaires tend to exhibit lower signal-to-noise ratios, and interactions with the stimulus material seem likely

To investigate this design feature, we grouped the filler tasks in the reanalyzed studies into three broad categories: verbal tasks (e.g., multiple-choice questions about short texts), numerical tasks (e.g., number puzzles, judging the correctness of math equations), and questionnaires (i.e., demographic measures or personality inventories). Figure 12 illustrates how signal-to-noise ratios vary across these categories in same-session designs. At first glance, verbal filler tasks show higher signal-to-noise ratios (*Mdn* =0.63) than numerical tasks (*Mdn* =0.55), with both outperforming questionnaires (*Mdn* =0.15). However, the lower signal-to-noise ratios observed in the numerical category largely stem from datasets that involved student samples or truth judgments in Phase 1, both factors we identified as detrimental to psychometric goodness. Excluding these conditions, verbal and numerical filler tasks exhibit comparable signal-to-noise ratios (*Mdn* =0.63 for both), whereas they remain consistently low for questionnaires (*Mdn* =0.15). This pattern suggests that the cognitive engagement elicited by the filler task may be more important than its specific content, with verbally and numerically demanding tasks yielding better psychometric properties than comparatively undemanding questionnaires.

At the same time, variation within the questionnaire category indicates that the filler task does not influence signal-to-noise ratios in isolation. Specifically, the differences in γ among these studies are associated with differences in the stimulus material: Studies with lower psychometric goodness used trivia statements combining known and unknown content, whereas those with higher psychometric goodness used trivia statements of uncertain validity. More generally, this example illustrates a central challenge of the present review: design characteristics in truth-effect experiments are unlikely to exert only independent effects. Instead, their influence on psychometric goodness may depend on how they are combined with other characteristics of the paradigm.

**Fig. 13:**
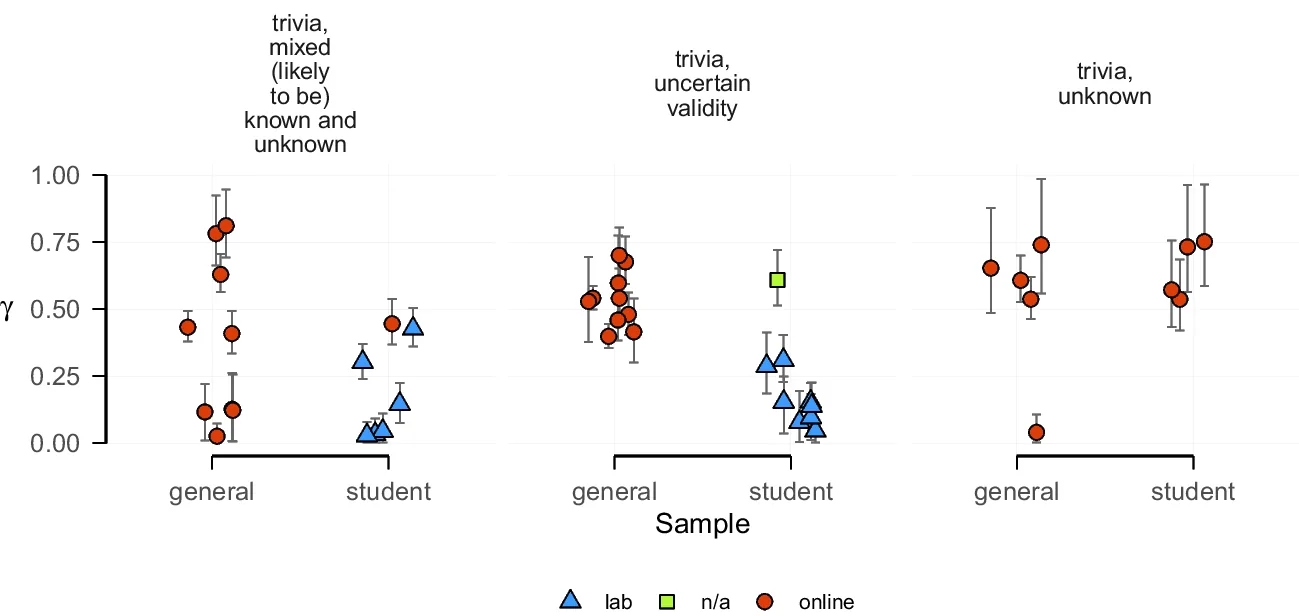
Interactions between characteristics. Signal-to-noise ratios (γ) are displayed by sample and stimulus material across studies. *Error bars* represent the 95% credible intervals

### Interactions between design characteristics

In light of the patterns described above, it appears highly plausible that different design features interact in their impact on psychometric goodness. Unfortunately, a comprehensive quantitative analysis of interactions is not feasible in the present analysis. Design characteristics are unevenly and non-orthogonally distributed across studies, resulting in very small cell sizes for many combinations (e.g., between 1 and 7 datasets for initial task × retention interval, and between 2 and 6 for filler task × sample). Therefore, we restrict ourselves to an exemplary descriptive exploration of one possible interaction using visualizations.

Figure 13 shows signal-to-noise ratios stratified by stimulus material and sample, reduced to the categories of stimuli where there are data for both general-population and student samples. At first glance, the pattern suggests an interaction between stimulus material and sample: student samples exhibit lower signal-to-noise ratios than general samples for mixed and uncertain trivia, whereas this difference disappears for unknown stimuli. The picture becomes more complex, however, once study setting is taken into account. Because studies using unknown trivia were conducted exclusively online, the observed pattern may instead reflect an interaction between stimulus material and study setting. Thus, this example illustrates not only the importance of considering interactions among design features, but also the difficulty of disentangling such interactions when design choices are correlated.

### Summary and recommendations

Building on these observations, we now summarize key insights and outline practical recommendations for optimizing the design of truth-effect experiments. Our analyses show that the choices of initial task during Phase 1, stimulus material, sample, study setting, retention interval, and filler task may have a substantial influence on the psychometric goodness of truth-effect studies. Once again, we caution readers not to interpret our findings as evidence of a causal relationship; we merely report associations observed in a retrospective analysis as a useful basis for planning future research on individual differences in the truth effect.

First, truth judgments administered during Phase 1 appear to engage different cognitive processes than those typically underlying the truth effect. We have found truth judgments as an initial task to be associated with decreased variability between individuals, whereas other tasks did not seem to influence psychometric goodness. We therefore recommend refraining from using truth judgments in Phase 1 and administering an unrelated task instead, for example, a categorization task or interest ratings.

Second, we recommend using unknown trivia statements as item material, as we have found these to be associated with reduced unsystematic trial noise and increased variability between individuals, resulting in larger signal-to-noise ratios.

Third, researchers should collect samples from the general population – rather than relying solely on student samples – to maximize the variability between individuals and, thus, the signal-to-noise ratio. If a student sample is necessary, deploying the study online may help broaden the participant pool and again increase variability between individuals.

Finally, we found variability between individuals to be more pronounced following shorter retention intervals with cognitively engaging filler tasks. Thus, we recommend short, same-session retention intervals filled with a verbally or numerically demanding task for studying individual differences, unless the research question specifically addresses the role of delay.

In sum, we recommend the following experimental design choices to maximize psychometric goodness when studying individual differences in the truth effect:

- **Initial task:** Categorization or interest ratings (no truth judgments);
- **Stimulus material:** Unknown trivia statements;
- **Sample:** General population;
- **Study setting:** Online;
- **Retention interval:** Short, within a single session;
- **Filler task:** Verbally or numerically demanding task (no questionnaires).

Importantly, these recommendations do not eliminate the need for adequate trial sizes. Without a sufficient number of observations per participant, even well-constructed experiments may yield unreliable results. Both psychometric goodness and trial size need to be sufficiently high to localize individual differences and interpret correlations with other variables. To calculate the required number of trials per person and condition for a given signal-to-noise ratio and desired reliability *r*, Eq. 3 may be solved for trial size *L*, yielding (Rouder et al., 2026)

$$\begin{aligned} L = \frac{\frac{2}{\gamma ^2}}{\frac{1}{r} - 1}. \end{aligned}$$

## Discussion

In this work, we present a structured, large-scale analysis of the psychometric goodness – as measured by the signal-to-noise ratio γ – of several published and unpublished truth-effect datasets. We first provided a comprehensive overview of overall psychometric goodness and then examined how it systematically varies across key design choices of truth-effect experiments such as the initial task, retention interval, filler task, sample, study setting, and stimulus material. While we observed low psychometric goodness in some of the analyzed datasets, it was quite high in others. Overall, this indicates that, under appropriate conditions, it is indeed possible to detect and interpret individual differences in the truth effect and correlations with other variables without relying on massively repeated trials.

Based on this overview, we derived empirically informed recommendations for designing truth-effect experiments with high psychometric goodness. Specifically, we found high psychometric goodness in studies that drew from general population samples, were conducted online, avoided truth judgments during Phase 1, used unknown trivia statements as item material, and implemented short, within-session retention intervals with cognitively demanding filler tasks. Although these insights and our recommendations are based on retrospective and descriptive analyses, they provide a valuable basis for future research aiming to examine individual differences in the truth effect more reliably.

Furthermore, our findings suggest that the ambiguous findings on individual differences in the truth effect reported in previous research may, at least in part, reflect methodological issues. Psychometric goodness varied considerably across the analyzed studies and there is no reason to assume a different state of affairs in the collection of studies that explicitly investigated individual differences in the truth effect – even though we could not include these studies in our analysis. Trial sizes also varied considerably. Taken together, we can expect that the reliability of individual truth-effect estimates is inconsistent across studies. While some may have had sufficient reliability to localize correlations, others may not. Consequently, we believe that the ambiguity in previous research reflects statistical rather than substantive issues, and that these issues can be overcome by carefully considering all design choices as outlined above.

In this context, we also want to draw attention to an important distinction between two closely related but conceptually distinct constructs: reliability and temporal stability. Temporal stability refers to the extent to which a psychological construct remains constant within individuals over time. Psychological traits, for example, are temporally stable constructs that are typically supposed to not change across measurement occasions. Reliability, in contrast, is a psychometric property of a measure that reflects the degree to which variability in the measurement reflects true variability in the measured construct. If a measure exhibits high reliability, this does not mean that it measures a temporally stable construct. Nevertheless, reliability is often operationalized as a test–retest correlation, which assumes that the measured construct is stable across test occasions. If that assumption does not hold, however, test–retest correlations may conflate true changes in the trait with low measurement reliability. In this sense, temporal stability is a critical prerequisite for meaningfully assessing test–retest reliability. Conversely, a reliable measure does not imply that there must be a high test–retest correlation. Consequently, for a meaningful assessment of individual differences, temporal stability and reliability must be considered separately, as they represent different, yet equally important concepts.

With this conceptual distinction as background, we now turn to the limited empirical evidence available on the temporal stability of the truth effect. To our knowledge, there has been only one attempt to directly investigate this question. Calio et al. (2025) report a weak to nonexistent test–retest correlation between two measurements of the truth effect spaced one week apart. However, their experimental design included only L=20 replicate trials per person and condition. Even though we do not have access to the raw data to evaluate the signal-to-noise ratio, with a trial size so small, we strongly suspect that the observed correlations may have been attenuated by low reliability. Along similar lines, Calio et al. (2025) concluded that while it may be possible that the truth effect is simply not temporally stable, an alternative explanation is that the truth-effect paradigm does not afford sufficient reliability to localize individual differences and correlations. Although we do not agree with the generality of this explanation, we strongly endorse the implication to carefully consider the psychometric properties of the employed experimental paradigms before drawing substantive conclusions. Crucially, the recommendations derived from our analysis now offer a solid ground to design truth-effect experiments that afford the necessary psychometric goodness and trial size to localize correlations between two or more measurement occasions such that valid conclusions about the temporal stability of the truth effect can be drawn.

### Limitations and future directions

An obvious limitation of our work is that our conclusions and recommendations are retrospective and descriptive. Many of the examined design choices are collinear (e.g., type of sample and study setting), which prevents a critical test of the unique influences of these choices. Furthermore, the specific levels of the experimental characteristics that we investigated were often influenced by subjective decisions. For instance, categorization of the stimulus material depended largely on how the authors of the original studies reported their methods. Therefore, we cannot infer any causal relationships between certain design choices and psychometric goodness from our results.

We sought to mitigate this limitation through transparent reporting and by building on the careful documentation provided by the original study authors. Nevertheless, future research should experimentally manipulate select design choices to quantify their unique impact on psychometric goodness. In some cases, we identified existing studies in this research that included such manipulations; for example, one study that systematically varied the initial task while keeping everything else constant (Zajdler, 2021). Nevertheless, a more systematic experimental investigation together with critical statistical tests of the unique effects of certain design choices on psychometric goodness would allow for clearer causal inferences.

In our review, we focused on a targeted set of experimental design choices. However, other potentially influential factors warrant further investigation in future research, such as the response scale, the number of repetitions, and verbatim versus non-verbatim repetition. We did not assess these factors due to limited variability in our selection of studies, but will provide some context in the following paragraphs. The choice of response scale on which participants provide their truth judgments might affect the signal-to-noise ratio. Most of our selected studies employed 5-, 6-, or 7-point Likert scales; however, it is conceivable that alternative formats, such as visual analog scales or sliders, could influence trial-level noise by increasing response granularity. On the other hand, prior psychometric research suggests that Likert scales and sliders have largely equivalent measurement properties (García-Pérez, 2023; Kuhlmann et al., 2017), and that increasing the number of response options beyond six offers no meaningful psychometric benefit (Simms et al., 2019). Consequently, it is also plausible that the choice of scale format may have minimal impact on psychometric goodness in truth-effect paradigms. Thus, it remains an open empirical question and a promising avenue for future research.

Another potentially relevant factor for the psychometric goodness of truth-effect studies is the number of repetitions of a statement prior to the truth judgment. The vast majority of studies rely on a single repetition, with truth judgments given at the second encounter (Henderson et al., 2022). However, prior work shows that the truth effect increases logarithmically with repeated exposure (Arkes et al., 1991; DiFonzo et al., 2016; Hassan and Barber, 2021; Hawkins et al., 2001). Even though the highest increase in perceived truth indeed happens at the second encounter, additional repetitions can still yield significant increases in perceived truthfulness (Hassan and Barber, 2021). Importantly, repetition effects are strongest when exposures are spaced rather than massed (Udry et al., 2022). Thus, to the extent that larger average effects are expected to be associated with greater between-individual variability, designs incorporating multiple spaced repetitions may afford higher signal-to-noise ratios.

Moreover, the present analysis did not investigate how verbatim as opposed to non-verbatim repetitions relate to signal-to-noise ratios. Assessing the impact of paraphrasing is challenging because, whereas verbatim repetition is clearly defined, gist repetitions or paraphrases may vary substantially in the degree to which they deviate from the original statement. Prior research shows that the truth effect extends beyond verbatim repetition to paraphrases, implications, and semantically related statements (Hawkins et al., 2001; Unkelbach and Rom, 2017; Vogel et al., 2020). However, few studies directly compare these formats to verbatim repetition. Available evidence suggests that modest paraphrasing of trivia statements (Silva et al., 2017) and naturally occurring reformulation of news headlines, where participants were exposed to different reformulations already during the exposure phase (Pillai and Fazio, 2025), yield truth effects comparable to verbatim repetition. In contrast, only loosely related statements tend to produce no truth effect (Udry and Barber, 2023). Together, these findings suggest that the truth effect emerges with repetition regardless of how an idea is expressed, provided the repeated statement remains semantically coherent with the original (Pillai and Fazio, 2025; Udry and Barber, 2023). Thus, whether non-verbatim repetition allows high psychometric goodness likely also depends on how strongly the repeated information can be linked to the original statement. Systematic investigations with targeted manipulations of repetition format, alongside response scale and repetition frequency, therefore represent important goals for future research.

In the present review, we largely considered design choices in isolation. In practice, however, these choices are likely interdependent and may jointly shape psychometric goodness in truth-effect experiments. A systematic analysis of such interactions was not feasible here because design characteristics were not independently manipulated and many combinations are sparsely represented across studies. We therefore restricted ourselves to an illustrative example, which highlights both the plausibility of interactions and the interdependency of design choices. Nonetheless, interactions are theoretically well motivated. For example, both the initial task and the retention interval independently influence psychometric goodness, making joint effects likely if initial tasks differentially influence encoding processes across retention intervals. Thus, systematic investigation of such interactions remains an important direction for future research, and will require studies that orthogonally manipulate key design characteristics within the same experimental framework to directly assess their unique as well as their joint impact on psychometric goodness.

A further limitation of this analysis is that, although this work draws on a large selection of published and unpublished datasets, it does not constitute an exhaustive review. The study selection was constrained by data availability, and the descriptive nature of our analyses does not allow for the quantification of possible biases resulting from this constraint. Nevertheless, the included data span a wide range of prominent design choices commonly used in truth-effect experiments and include a sufficient number of data points to support meaningful conclusions about designing good truth-effect experiments.

Going forward, we strongly encourage researchers to routinely calculate and report signal-to-noise ratios in their experiments. Doing so would not only enable researchers to immediately evaluate the psychometric properties of their specific experiment and the quality of their data, but it would also expand the empirical basis for understanding the impact of certain design choices. To facilitate the calculation in practice, we explicitly refer to the method-of-moments estimator proposed by Rouder and Mehrvarz (2024) that allows for straightforward computation of signal-to-noise ratios from standard statistics, without the need for more sophisticated hierarchical, Bayesian modeling.

Finally, we want to emphasize that our work is not a call to standardize all truth-effect research. We want to make researchers aware of the consequences certain design choices in their experiments may have on the variability between individuals, trial noise, and effect sizes. For those studies aiming to investigate individual differences in the truth effect, we provide practical guidelines to increase psychometric goodness – and, ultimately, reliability – for localizing individual truth effects and correlations with other psychological variables. Nevertheless, manipulating experimental design factors remains important to investigate how situational factors such as context, warnings, depth of processing at exposure, and source credibility, to name a few, influence the effect of repetition on people’s evaluation of truth.

### Conclusion

This systematic review of published and unpublished datasets advances our understanding of the psychometric properties of truth-effect experiments and provides practical recommendations for future research. Our analysis reveals substantial variation in psychometric goodness across studies, as measured by the signal-to-noise ratio γ. While some experiments exhibit low γ values, others demonstrate considerably higher values – indicating that, in principle, reliable measurement of individual differences in the truth effect and their correlations with other variables is possible. However, low signal-to-noise ratios and insufficient trial sizes have likely contributed to the inconsistent findings on individual differences observed in previous work. To address this issue, we identify several design features that influence psychometric goodness and offer concrete recommendations on how to optimize them. We further encourage researchers to routinely report signal-to-noise ratios and trial sizes, thereby increasing transparency and enabling more accurate evaluation of the psychometric quality of experiments. Taken together, our findings aim to put experimental research on individual differences in the truth effect on firmer methodological ground to support the development of more robust, generalizable insights into the individual mechanisms underlying the truth effect.

## Data Availability

This study is based on a reanalysis of existing datasets. We do not hold the rights to publish data collected by other authors; however, all data are publicly available or may be obtained from the original authors. For all datasets, we provide references to the original data sources and, where necessary, author contact information in the appendix (Table 2).

## Appendix A Models and prior specification

Let $Y_{ij\ell }$ denote the truth judgment made by individual i=1,...,I for condition j=1 (novel) or j=2 (repeated), on trial ℓ=1,...,L. We specified a hierarchical linear model with person-specific intercepts and slopes:

$$\begin{aligned} Y_{ij\ell } \sim \mathcal {N}(\alpha _i + x_j \theta _i, \sigma ^2_W), \end{aligned}$$

where $\alpha _i$ is the *i*th individual’s average truth judgment, $x_j = -0.5, 0.5$ is the contrast-coded repetition condition, and $\theta _i$ is the *i*th individual’s true effect of repetition. The component $\sigma ^2_W$ reflects the unsystematic trial-by-trial variability (or noise) within each individual. To complete the hierarchical model specification, we place random-effects models on the person-specific parameters:

$$\begin{aligned} \alpha _i \sim \mathcal {N}(\mu , \sigma ^2), \end{aligned}$$

where μ is the average truth rating across individuals (i.e., the grand-mean intercept) and σ is the variability in average truth judgments between individuals;

$$\begin{aligned} \theta _i \sim \mathcal {N}(\nu , \sigma ^2_B), \end{aligned}$$

where ν is the average truth effect in the population and $\sigma _B$ the systematic variability between individuals.

Model analyses were conducted using the brms package (Bürkner, 2017), which provides an interface for fitting Bayesian generalized linear multilevel models in R with Stan. All model parameters were estimated using the broad default priors implemented in brms. These correspond to a flat prior for fixed slopes, indicating no prior preference for any particular effect size:

$$\begin{aligned} \nu \propto 1, \end{aligned}$$

a weakly informative Student-t prior for the fixed intercept:

$$\begin{aligned} \mu \sim \text {Student-t}(3, 0.3,2.5), \end{aligned}$$

and weakly-informative Student-t priors for the standard deviations:

$$\begin{aligned} \sigma _W \sim \text {Student-t}(3, 0, 2.5), \\ \sigma _B \sim \text {Student-t}(3, 0, 2.5), \\ \sigma \sim \text {Student-t}(3, 0, 2.5). \end{aligned}$$

We ran eight Markov chains with 3000 iterations each, discarding 1000 warm-up iterations per chain, resulting in a total of 16, 000 post-warm-up samples. Sampling was performed using the No-U-Turn Sampler (NUTS), the default algorithm in brms. All analysis scripts, including data preparation steps, are available on the Open Science Framework (https://osf.io/me367).

The extended model incorporates both person- and item-level effects. Truth judgments $Y_{ijk\ell }$ are modeled as:

$$\begin{aligned} Y_{ijk\ell } \sim \mathcal {N}(\alpha _i + t_k \beta _i + x_j \theta _i + \eta _k, \sigma ^2_W), \end{aligned}$$

where $\alpha _i$ is the person-specific intercept, $\beta _i$ and $\theta _i$ are subject-specific slopes for item truth status ($t_k = 0, 1$ for false versus true statements) and repetition condition ($x_j = -0.5, 0.5$ for new versus repeated statements), respectively, and $\eta _k$ is a random intercept for item *k*, capturing item-specific variation in baseline truth judgments. We place random-effect models on the person-specific intercept and slopes:

$$\begin{aligned} \alpha _i&\sim \mathcal {N}(\mu , \sigma ^2), \\ \beta _i&\sim \mathcal {N}(\delta , \tau ^2), \\ \theta _i&\sim \mathcal {N}(\nu , \sigma ^2_B), \end{aligned}$$

and on the item-specific intercept:

$$\begin{aligned} \eta _k \sim \mathcal {N}(0, \kappa ^2). \end{aligned}$$

We again rely on the default brms priors. The population-level slopes δ and ν have flat, uninformative priors:

$$\begin{aligned} \delta&\propto 1,\\ \nu&\propto 1. \end{aligned}$$

The overall intercept μ and all standard deviations have mildly informative Student-t priors:

$$\begin{aligned} \mu&\sim \text {Student-t}(3, 0.2, 2.5),\\ \tau&\sim \text {Student-t}(3, 0, 2.5), \\ \sigma _B&\sim \text {Student-t}(3, 0, 2.5), \\ \sigma _W&\sim \text {Student-t}(3, 0, 2.5), \\ \kappa&\sim \text {Student-t}(3, 0, 2.5). \end{aligned}$$

## Appendix B Data availability

**Table 2:** Data Sources

Report	Data Source
Brashier et al. (2020) Exp. 1	https://osf.io/bxuj2
Brashier et al. (2020) Exp. 3	https://osf.io/vqtw5
Brashier et al. (2020) Exp. 4	https://osf.io/2bk7f
Cubeddu et al. (2024)	https://osf.io/xtduc
De Keersmaecker et al. (2026) Exp. 1	https://osf.io/ang82?view_only=6c9b8bd534644bc382849aaea9e153ee
De Keersmaecker et al. (2026) Exp. 2	https://osf.io/pm83e?view_only=6c9b8bd534644bc382849aaea9e153ee
Effron and Raj (2020)	Author Contact: deffron@london.edu
Hatzidaki et al. (2025)	https://osf.io/7vz6w
Henderson et al. (2021)	https://osf.io/2aenr
Jalbert et al. (2020) Exp. 1	https://osf.io/jznr2
Jalbert et al. (2020) Exp. 2	https://osf.io/24wkv
Jalbert et al. (2020) Exp. 3	https://osf.io/3w6vz
Lacassagne et al. (2022)	https://osf.io/2pgmx
Lorenzoni et al. (2024)	https://osf.io/zc9n7
Murray et al. (2020)	https://osf.io/4nsrx
Nadarevic (2007)	https://osf.io/97g4j
Nadarevic and Rinnewitz (2011)	https://osf.io/jfz25
Nadarevic et al. (2012)	https://osf.io/cvpmr
Nadarevic and Erdfelder (2014) Exp. 1	https://osf.io/qsrw5
Nadarevic and Erdfelder (2014) Exp. 2	https://osf.io/amu4w
Nadarevic and Erdfelder (2025) Exp. 1	https://osf.io/teudp
Nadarevic and Erdfelder (2025) Exp. 2	https://osf.io/5wxju
Nadarevic and Aßfalg (2017) Exp. 1	https://osf.io/4znvx
Nadarevic and Aßfalg (2017) Exp. 2	https://osf.io/qz2rg
Nadarevic et al. (2018)	https://osf.io/4a56u
Newman et al. (2020)	https://data.mendeley.com/datasets/r68dcdjrpc/1
Pennycook et al. (2018)	https://osf.io/tc65a
Unkelbach and Speckmann (2021)	https://osf.io/pjhf6
Vogel et al. (2020)	https://osf.io/mtpf3
Zajdler (2021)	https://osf.io/sygwb

Note. Reports and respective data sources

## Footnotes

1 Note that the signal-to-noise ratio is generally lower in Nadarevic and Erdfelder (2014) because truth judgments were administered as an initial task during Phase 1.

## Code Availability

R code to reproduce all analyses, figures, and tables is available on the Open Science Framework: https://osf.io/me367

## References

[CR1] Arkes, H. R., Boehm, L. E., & Xu, G. (1991). Determinants of judged validity. *Journal of Experimental Social Psychology,**27*(6), 576–605. 10.1016/0022-1031(91)90026-3

[CR2] Boehm, L. E. (1994). The validity effect: A search for mediating variables. *Personality and Social Psychology Bulletin,**20*(3), 285–293. 10.1177/0146167294203006

[CR3] Brashier, N. M., Eliseev, E. D., & Marsh, E. J. (2020). An initial accuracy focus prevents illusory truth. *Cognition,**194*, Article 104054. 10.1016/j.cognition.2019.104054 31473395

[CR4] Brashier, N. M., Umanath, S., Cabeza, R., & Marsh, E. J. (2017). Competing cues: Older adults rely on knowledge in the face of fluency. *Psychology and Aging,**32*(4), 331–337. 10.1037/pag0000156 PMC5476227 28333505

[CR5] Bürkner, P.-C. (2017). Brms: An R package for Bayesian multilevel models using stan. *Journal of Statistical Software,**80*(1), 1–28. 10.18637/jss.v080.i01

[CR6] Calio, F., Nadarevic, L., & Musch, J. (2025). Assessing the truth effect’s reliability and test–retest stability. *Consciousness and Cognition,**135*, Article 103923. 10.1016/j.concog.2025.103923 40876151

[CR7] Cubeddu, V., Béna, J., & Corneille, O. (2024). Are there stable individual qualitative differences in the truth effect? Unpublished raw data. Retrieved from https://osf.io/3ax97

[CR8] De Keersmaecker, J., Dunning, D., Pennycook, G., Rand, D. G., Sanchez, C., Unkelbach, C., & Roets, A. (2020). Investigating the robustness of the illusory truth effect across individual differences in cognitive ability, need for cognitive closure, and cognitive style. *Personality and Social Psychology Bulletin,**46*(2), 204–215. 10.1177/0146167219853844 31179863

[CR9] De Keersmaecker, J., Wiernik, B. M., Roets, A., & Unkelbach, C. (2026). *Truth by repetition reliably differs between people over time*. PsyArXiv. 10.31234/osf.io/aeukr_v2

[CR10] Dechêne, A., Stahl, C., Hansen, J., & Wänke, M. (2010). The truth about the truth: A meta-analytic review of the truth effect. *Personality and Social Psychology Review,**14*(2), 238–257. 10.1177/1088868309352251 20023210

[CR11] DiFonzo, N., Beckstead, J. W., Stupak, N., & Walders, K. (2016). Validity judgments of rumors heard multiple times: The shape of the truth effect. *Social Influence,**11*(1), 22–39. 10.1080/15534510.2015.1137224

[CR12] Ecker, U. K. H., Lewandowsky, S., Cook, J., Schmid, P., Fazio, L. K., Brashier, N., & Amazeen, M. A. (2022). The psychological drivers of misinformation belief and its resistance to correction. *Nature Reviews Psychology,**1*(1), 13–29. 10.1038/s44159-021-00006-y

[CR13] Effron, D. A., & Raj, M. (2020). Misinformation and morality: Encountering fake-news headlines makes them seem less unethical to publish and share. *Psychological Science,**31*(1), 75–87. 10.1177/0956797619887896 31751517

[CR14] Fanny, C., & Istiono, W. (2022). Analysis timeline user content on Instagram using simple additive weighting algorithm. *Journal of Applied Computer Science & Mathematics,**16*(1), 14–17. 10.4316/JACSM.202201002

[CR15] Fazio, L. K., Brashier, N. M., Payne, B. K., & Marsh, E. J. (2015). Knowledge does not protect against illusory truth. *Journal of Experimental Psychology: General,**144*(5), 993–1002. 10.1037/xge0000098 26301795

[CR16] Fazio, L. K., Pillai, R. M., & Patel, D. (2022). The effects of repetition on belief in naturalistic settings. *Journal of Experimental Psychology: General,**151*(10), 2604–2613. 10.1037/xge0001211 35286116

[CR17] Fazio, L. K., & Sherry, C. L. (2020). The effect of repetition on truth judgments across development. *Psychological Science,* *31*(9), 1150–1160. 10.1177/0956797620939534 32857670

[CR18] Garcia-Marques, T., Silva, R. R., & Mello, J. (2016). Judging the truth-value of a statement in and out of a deep processing context. *Social Cognition,**34*(1), 40–54. 10.1521/soco.2016.34.1.40

[CR19] García-Pérez, M. A. (2023). Are the steps on Likert scales equidistant? Responses on visual analog scales allow estimating their distances. *Educational and Psychological Measurement,**84*(1), 91–122. 10.1177/00131644231164316 PMC10795572 38250504

[CR20] Green, S. B., Yang, Y., Alt, M., Brinkley, S., Gray, S., Hogan, T., & Cowan, N. (2016). Use of internal consistency coefficients for estimating reliability of experimental task scores. *Psychonomic Bulletin & Review,**23*(3), 750–763. 10.3758/s13423-015-0968-3 PMC5484005 26546100

[CR21] Haaf, J. M., Hoffstadt, M., & Lesche, S. (2025). Attentional control data collection: A resource for efficient data reuse. *Behavior Research Methods*, *57*(8), Article 208. 10.3758/s13428-025-02717-z PMC12187800 40555897

[CR22] Hasher, L., Goldstein, D., & Toppino, T. (1977). Frequency and the conference of referential validity. *Journal of Verbal Learning and Verbal Behavior,**16*(1), 107–112. 10.1016/S0022-5371(77)80012-1

[CR23] Hassan, A., & Barber, S. J. (2021). The effects of repetition frequency on the illusory truth effect. *Cognitive Research: Principles and Implications,**6*(1), Article 38. 10.1186/s41235-021-00301-5 PMC8116821 33983553

[CR24] Hatzidaki, A., Santesteban, M., & Navarrete, E. (2025). Illusory truth effect across languages and scripts. *Psychonomic Bulletin & Review,**32*(3), 1231–1239. 10.3758/s13423-024-02596-z 39466589

[CR25] Hawkins, S. A., Hoch, S. J., & Meyers-Levy, J. (2001). Low-Involvement Learning: Repetition and Coherence in Familiarity and Belief. *Journal of Consumer Psychology,**11*(1), 1–11. 10.1207/S15327663JCP1101_1

[CR26] Henderson, E. L., Simons, D. J., & Barr, D. J. (2021). The trajectory of truth: A longitudinal study of the illusory truth effect. *Journal of Cognition,**4*(1), Article 29. 10.5334/joc.161 PMC8194981 34164597

[CR27] Henderson, E. L., Westwood, S. J., & Simons, D. J. (2022). A reproducible systematic map of research on the illusory truth effect. *Psychonomic Bulletin & Review,**29*(3), 1065–1088. 10.3758/s13423-021-01995-w PMC9166874 34708397

[CR28] Jalbert, M., Schwarz, N., & Newman, E. (2020). Only half of what i’ll tell you is true: Expecting to encounter falsehoods reduces illusory truth. *Journal of Applied Research in Memory and Cognition,**9*(4), 602–613. 10.1016/j.jarmac.2020.08.010

[CR29] Kuhlmann, T., Dantlgraber, M., & Reips, U. D. (2017). Investigating measurement equivalence of visual analogue scales and Likert-type scales in internet-based personality questionnaires. *Behavior Research Methods,**49*, 2173–2181. 10.3758/s13428-016-0850-x 28130728

[CR30] Lacassagne, D., Béna, J., & Corneille, O. (2022). Is Earth a perfect square? Repetition increases the perceived truth of highly implausible statements. *Cognition,**223*, Article 105052. 10.1016/j.cognition.2022.105052 35144111

[CR31] Le Bon, G. (1895). *The crowd: A study of the popular mind*. [Released 2003]. Whitefish, MT: Kessinger Publishing. Retrieved from https://coronacircus.com/wp-content/uploads/2020/04/Gustave-Le-Bon-TheCrowd_-A-Study-of-the-Popular-Mind-1982.pdf

[CR32] Lin, H., Savio, M. T., Huang, X., Steiger, M., Guevara, R. L., Szostak, D.,... Rand, D. G. (2024). Accuracy prompts protect professional content moderators from the illusory truth effect. *PNAS Nexus*, *3*(11), Article 481. 10.1093/pnasnexus/pgae481 PMC11574866 39564570

[CR33] Lorenzoni, A., Faccio, R., & Navarrete, E. (2024). Does foreign-accented speech affect credibility? Evidence from the illusory-truth paradigm. *Journal of Cognition,**7*(1), Article 26. 10.5334/joc.353 PMC10885845 38405636

[CR34] Murray, S., Stanley, M., McPhetres, J., Pennycook, G., & Seli, P. (2020). "I’ve said it before and I will say it again": Repeating statements made by Donald Trump increases perceived truthfulness for individuals across the political spectrum. PsyArXiv. 10.31234/osf.io/9evzc

[CR35] Nadarevic, L. (2007). A failed replication of the truth effect. Unpublished raw data. 10.17605/OSF.IO/FW2QE

[CR36] Nadarevic, L., & Aßfalg, A. (2017). Unveiling the truth: Warnings reduce the repetition-based truth effect. *Psychological Research,**81*(4), 814–826. 10.1007/s00426-016-0777-y 27318939

[CR37] Nadarevic, L., & Erdfelder, E. (2014). Initial judgment task and delay of the final validity-rating task moderate the truth effect. *Consciousness and Cognition,**23*, 74–84. 10.1016/j.concog.2013.12.002 24370608

[CR38] Nadarevic, L., & Erdfelder, E. (2025). On the relationship between recognition judgments and truth judgments: Memory states moderate the recognition-based truth effect. *Journal of Experimental Psychology: Learning, Memory, and Cognition,**51*(11), 1778–1795. 10.1037/xlm0001460 39964518

[CR39] Nadarevic, L., Meckler, D., & Schmidt, A. (2012). An investigation of the truth effect and different personality traits. Unpublished raw data. 10.17605/OSF.IO/6wv4z

[CR40] Nadarevic, L., Plier, S., Thielmann, I., & Darancó, S. (2018). Foreign language reduces the longevity of the repetition-based truth effect. *Acta Psychologica,**191*, 149–159. 10.1016/j.actpsy.2018.08.019 30273765

[CR41] Nadarevic, L., & Rinnewitz, L. (2011). Judgment mode instructions do not moderate the truth effect. Unpublished raw data. 10.17605/OSF.IO/3uaj7

[CR42] Newman, E. J., Jalbert, M. C., Schwarz, N., & Ly, D. P. (2020). Truthiness, the illusory truth effect, and the role of need for cognition. *Consciousness and Cognition,**78*, Article 102866. 10.1016/j.concog.2019.102866 31935624

[CR43] Orchinik, R., Rand, D. G., & Bhui, R. (2025). *The not so illusory truth effect: A rational foundation for repetition effects.* PsyArXiv. 10.31234/osf.io/qvwdy_v3

[CR44] Parks, C. M., & Toth, J. P. (2006). Fluency, familiarity, aging, and the illusion of truth. *Aging, Neuropsychology, and Cognition,**13*(2), 225–253. 10.1080/138255890968691 16807200

[CR45] Pennycook, G., Cannon, T. D., & Rand, D. G. (2018). Prior exposure increases perceived accuracy of fake news. *Journal of Experimental Psychology: General,**147*(12), 1865–1880. 10.1037/xge0000465 PMC6279465 30247057

[CR46] Peterson, R. A., & Merunka, D. R. (2014). Convenience samples of college students and research reproducibility. *Journal of Business Research,**67*(5), 1035–1041. 10.1016/j.jbusres.2013.08.010

[CR47] Pillai, R. M., & Fazio, L. K. (2021). The effects of repeating false and misleading information on belief. *WIREs Cognitive Science,**12*(6), Article e1573. 10.1002/wcs.1573 34423562

[CR48] Pillai, R. M., & Fazio, L. K. (2025). Repeated by many versus repeated by one: Examining the role of social consensus in the relationship between repetition and belief. *Journal of Applied Research in Memory and Cognition,**14*(2), 154–166. 10.1037/mac0000166

[CR49] Pillai, R. M., Fazio, L. K., & Effron, D. A. (2023). Repeatedly encountered descriptions of wrongdoing seem more true but less unethical: Evidence in a naturalistic setting. *Psychological Science,**34*(8), 863–874. 10.1177/09567976231180 578 37428445

[CR50] R Core Team. (2025). R: A language and environment for statistical computing. *R Foundation for Statistical Computing*. 10.32614/R.manuals

[CR51] Riesthuis, P., & Woods, J. (2024). “That’s just like, your opinion, man”: The illusory truth effect on opinions. *Psychological Research,**88*(1), 284–306. 10.1007/s00426-023-01845-5 PMC10257371 37300704

[CR52] Rouder, J. N., & Haaf, J. M. (2019). A psychometrics of individual differences in experimental tasks. *Psychonomic Bulletin & Review,**26*(2), 452–467. 10.3758/s13423-018-1558-y 30911907

[CR53] Rouder, J. N., Kumar, A., & Haaf, J. M. (2023). Why many studies of individual differences with inhibition tasks may not localize correlations. *Psychonomic Bulletin & Review,**30*(6), 2049–2066. 10.3758/s13423-023-02293-3 PMC10728261 37450264

[CR54] Rouder, J. N., & Mehrvarz, M. (2024). Hierarchical-model insights for planning and interpreting individual-difference studies of cognitive abilities. *Current Directions in Psychological Science,**33*(2), 128–135. 10.1177/09637214231220923

[CR55] Rouder, J. N., Mehrvarz, M., & Schnuerch, M. (2026). The role of reliability in experiments. *British Journal of Mathematical and Statistical Psychology*, Article bmsp.70042. 10.1111/bmsp.70042 41834697

[CR56] Schnuerch, M., Nadarevic, L., & Rouder, J. N. (2021). The truth revisited: Bayesian analysis of individual differences in the truth effect. *Psychonomic Bulletin & Review,**28*(3), 750–765. 10.3758/s13423-020-01814-8 PMC8219594 33104997

[CR57] Schnuerch, M., & Rouder, J. N. (2026). Assessing qualitative individual differences with Bayesian hierarchical latent-mixture models. *Psychological Methods*. Advance online publication. 10.1037/met0000840 42113111

[CR58] Silva, R. R., Garcia-Marques, T., & Reber, R. (2017). The informative value of type of repetition: Perceptual and conceptual fluency influences on judgments of truth. *Consciousness and Cognition,**51*, 53–67. 10.1016/j.concog.2017.02.016 28288382

[CR59] Simms, L. J., Zelazny, K., Williams, T. F., & Bernstein, L. (2019). Does the number of response options matter? Psychometric perspectives using personality questionnaire data. *Psychological Assessment,**31*(4), 557–566. 10.1037/pas0000648 30869956

[CR60] Spearman, C. (1904). The proof and measurement of association between two things. *The American Journal of Psychology,**15*(1), 72–101. 10.2307/1412159 3322052

[CR61] Stump, A., Rummel, J., & Voss, A. (2022). Is it all about the feeling? Affective and (meta-)cognitive mechanisms underlying the truth effect. *Psychological Research,**86*(1), 12–36. 10.1007/s00426-020-01459-1 PMC8821071 33484352

[CR62] Stump, A., Voss, A., & Rummel, J. (2024). The illusory certainty: Information repetition and impressions of truth enhance subjective confidence in validity judgments independently of the factual truth. *Psychological Research,**88*(4), 1288–1297. 10.1007/s00426-024-01956-7 PMC11143013 38526581

[CR63] Udry, J., & Barber, S. J. (2023). The illusory truth effect requires semantic coherence across repetitions. *Cognition,**241*, Article 105607. 10.1016/j.cognition.2023.105607 37742428

[CR64] Udry, J., White, S. K., & Barber, S. J. (2022). The effects of repetition spacing on the illusory truth effect. *Cognition,**225*, Article 105157. 10.1016/j.cognition.2022.105157 35533415

[CR65] Underwood, B. J. (1975). Individual differences as a crucible in theory construction. *American Psychologist,**30*(2), 128–134. 10.1037/h0076759

[CR66] Unkelbach, C., & Rom, S. C. (2017). A referential theory of the repetition-induced truth effect. *Cognition,**160*, 110–126. 10.1016/j.cognition.2016.12.016 28088712

[CR67] Unkelbach, C., & Speckmann, F. (2021). Mere repetition increases belief in factually true COVID-19-related information. *Journal of Applied Research in Memory and Cognition,**10*(2), 241–247. 10.1016/j.jarmac.2021.02.001

[CR68] Vellani, V., Zheng, S., Ercelik, D., & Sharot, T. (2023). The illusory truth effect leads to the spread of misinformation. *Cognition,**236*, Article 105421. 10.1016/j.cognition.2023.105421 PMC10636596 36871397

[CR69] Vogel, T., Silva, R. R., Thomas, A., & Wänke, M. (2020). Truth is in the mind, but beauty is in the eye: Fluency effects are moderated by a match between fluency source and judgment dimension. *Journal of Experimental Psychology: General,**149*(8), 1587–1596. 10.1037/xge0000731 31944810

[CR70] Weigold, A., & Weigold, I. K. (2022). Traditional and modern convenience samples: An investigation of college student, mechanical turk, and mechanical turk college student samples. *Social Science Computer Review,**40*(5), 1302–1322. 10.1177/08944393211006847

[CR71] Zajdler, S. (2021). *Explaining individual differences in the truth effect*. [Bachelor thesis]. University of Mannheim

